# Structure sampling for computational estimation of localized DNA interaction rates

**DOI:** 10.1038/s41598-021-92145-8

**Published:** 2021-06-16

**Authors:** Sarika Kumar, Julian M. Weisburd, Matthew R. Lakin

**Affiliations:** 1 Department of Computer Science, University of New Mexico, Albuquerque, NM 87131 USA; 2 Department of Chemical & Biological Engineering, University of New Mexico, Albuquerque, NM 87131 USA; 3 Center for Biomedical Engineering, University of New Mexico, Albuquerque, NM 87131 USA

**Keywords:** DNA computing, DNA nanostructures

## Abstract

Molecular circuits implemented using molecular components tethered to a DNA tile nanostructure have certain advantages over solution-phase circuits. Tethering components in close proximity increases the speed of reactions by reducing diffusion and improves scalability by enabling reuse of identical DNA sequences at different locations in the circuit. These systems show great potential for practical applications including delivery of diagnostic and therapeutic molecular circuits to cells. When modeling such systems, molecular geometry plays an important role in determining whether the two species interact and at what rate. In this paper, we present an automated method for estimating reaction rates in tethered molecular circuits that takes the geometry of the tethered species into account. We probabilistically generate samples of structure distributions based on simple biophysical models and use these to estimate important parameters for kinetic models. This work provides a basis for subsequent enhanced modeling and design tools for localized molecular circuits.

## Introduction

Molecular computing is a promising research area, as autonomous molecular devices could be used to sense their environment and activate a response based on complex information processing. They therefore have great potential in biomedical diagnostics^1^ and drug delivery^2^. These systems can be implemented using solution-phase or localized circuits. In solution-phase circuits, molecular components diffuse freely in solution which limits the interaction rates of the molecular species and increases the chance of crosstalk. Tethering molecular components to a surface accelerates computation as the interacting species do not have to diffuse closer in order to interact^3^. Such systems are also more scalable as identical sequences can be safely reused in different locations^4^. DNA origami^5^ has proven to be a reliable method for implementing spatially addressable nanostructures^6^, exploiting the sequence specificity of DNA hybridization to assemble strands into programmed nanoscale structures such as molecular computing circuits^4^. In this work, we study the problem of computationally designing tethered molecular circuits, in particular, the question of estimating reaction rates from a high-level description of circuit geometry.

**Figure 1:**
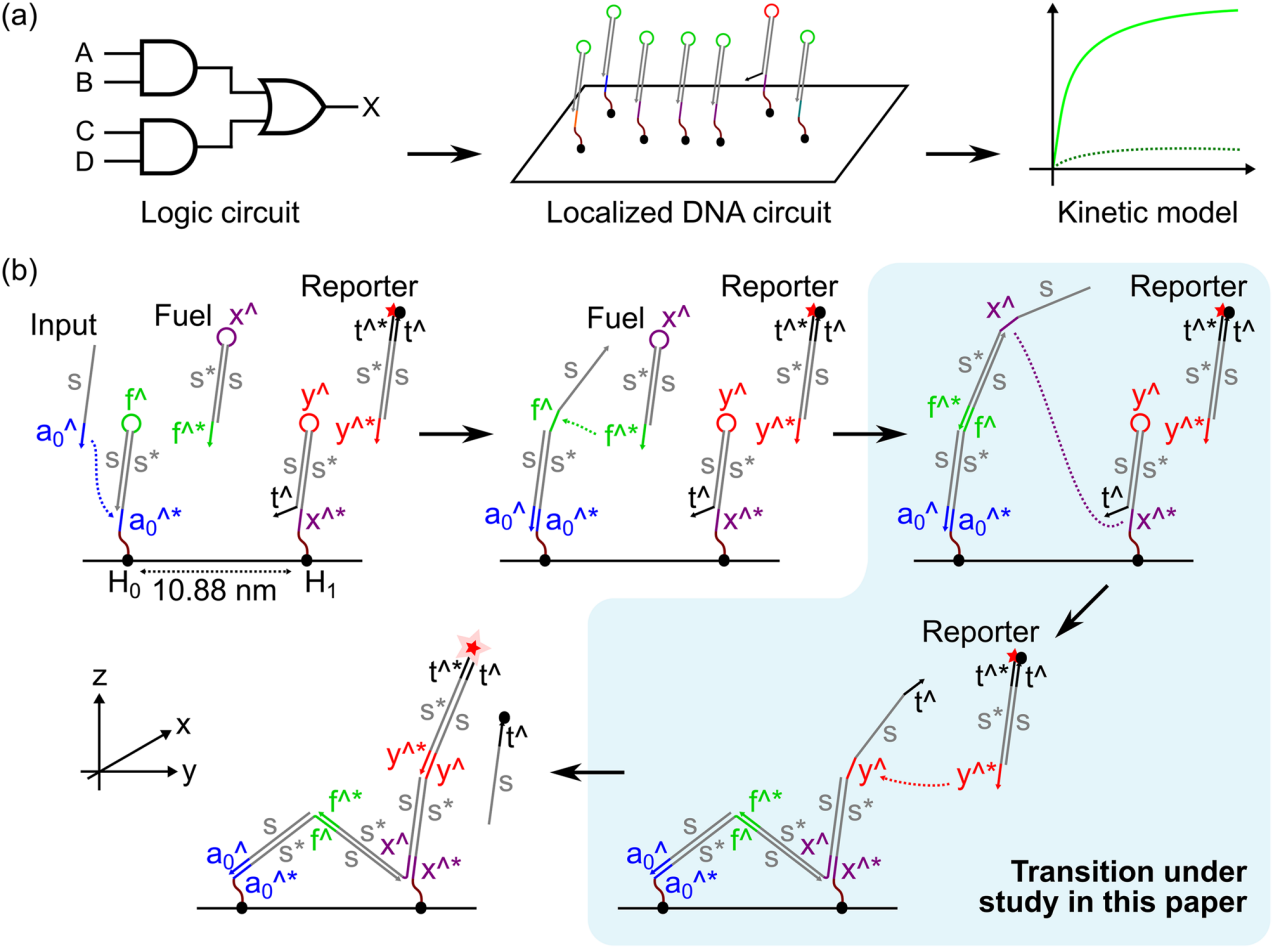
Outline of this work and the system under study. (**a**) Boolean logic circuits can be converted into localized DNA strand displacement cascades on the surface of a DNA origami tile, as previously demonstrated^4^. This paper concerns the automated conversion of such circuit designs into kinetic models by estimating reaction rate constants. (**b**) Cartoon of a two-hairpin signal transmission wire^4^. Binding of an input strand opens the first hairpin ($H_0$), enabling a fuel hairpin to bind to it. The opened fuel hairpin can then interact with the second hairpin ($H_1$), opening that too. The opened $H_1$ hairpin can then activate the reporter, producing a fluorescent output. The shaded blue area indicates the specific transition under study in this paper. Also shown are the directions of the axes that will be used throughout this paper.

Toehold-mediated DNA strand displacement (TMSD) reactions are a promising approach for implementing enzyme-free molecular circuits^7^. TMSD is a class of reactions in which an incoming strand of DNA displaces another strand that is initially bound to a third strand, with the binding nucleated by a short overhanging “toehold” domain^8,9^. Various DNA devices have been built using this mechanism like logic circuits^10–12^, molecular motors^8,13^, neural networks^14,15^, and catalytic signal amplifiers^16^. To effectively design such circuits, it is critical to understand the relevant reaction kinetics. Previously Zhang et al.^17^ demonstrated that the kinetics of strand displacement reactions can be modulated via the length of the toehold. The hybridization rate constant of an unknown sequence has also been predicted using a machine-learning model trained on similar reactions with experimentally measured rate constants^18^. Srinivas et al.^19^ estimated the kinetics of strand displacement reactions using both secondary structure modeling and also used a coarse-grained molecular model called oxDNA^20^. The oxDNA model incorporates biophysical details such as the geometric and steric effects of single and double stranded nucleic acids. However, all of the above work focuses on solution-phase molecular circuits; in this paper, we use simple computational modeling techniques to estimate similar rate constants for TMSD reactions localized to a surface.

Experimentally determining the kinetics of localized reactions is difficult because the components are not freely diffusing in the bulk solution, therefore they occur relatively rapidly. This makes it hard to accurately measure the rates of localized reactions. Therefore, in this work we take a computational approach to estimating these reaction rates. This approach is complicated by the fact that one cannot make many simplifying assumptions as can be done in the case of solution-phase circuits. In particular, in solution we can assume that interacting components can rotate and translate into whatever relative positioning is required for the reaction to proceed, and this is reflected in the value of the corresponding bimolecular rate constant. However, in localized circuits the conformations and motions of the reactants are restricted and we must therefore consider the molecular geometry of the interacting species in our models. In this paper, we probabilistically estimate the rates of localized toehold binding reactions by randomly sampling an ensemble of structures for each of the reactants, based on simple biophysical models that account for molecular geometry. We choose this approach because of its simplicity, which offers the potential for such computations to be run rapidly within an automated system for compiling structural models of DNA circuits into kinetic models. Indeed, one of us has previously published such a system^21^, though it lacked the ability to automatically infer rate constants that we demonstrate here.

Early theoretical work proposed DNA architectures for localized circuits^22,23^. More recently, Dalchau et al.^24^ analyzed the behavior of localized logic circuits using the chemical master equation, which can be applied due to the low number of total states in a localized system. That work aimed to approximate the kinetics of the studied localized reactions via the *local concentration* approach^25^. Briefly, this approach aims to scale the rate constant values for corresponding reactions in the solution-phase into appropriate rate constant values for the localized reaction, by multiplying by a factor which we refer to as the local concentration. We consider the rate constant for a localized reaction to be unimolecular because both “reactant” structures are tethered to the same underlying DNA origami nanostructure and therefore are not diffusing independently throughout a bulk solution. To see why this scaling is necessary, note that the unit of the rate constant of a bimolecular reaction is $M^{-1}s^{-1}$, whereas the unit of the rate constant of a unimolecular reaction is $s^{-1}$. Therefore, to convert from a bimolecular rate constant to a unimolecular one, one must multiply by a concentration. A helpful informal interpretation of this concentration is that it represents the apparent concentration of one reactant that would be required in bulk solution to produce the same overall rate of the reaction as is observed by the other reaction in the localized version of the reaction. The goal of this work is to computationally determine the local concentration from a specific localized reaction of interest, derived from previous work by Chatterjee et al.^4^.

In tethered molecular computing systems, components are attached to a surface and are therefore constrained in their physical location and orientation. In particular, the components may not interact if they are positioned too far apart or their orientation relative to each other is wrong. In previous work, we have tackled this problem by translating a tethered molecular circuit into a constraint problem that represents all the possible physical configurations of the molecular components and used satisfaction modulo theories (SMT) solving to automatically determine whether a given structural arrangement of the DNA domains is physically plausible^26^. However, that work only gives a binary “yes/no” answer on whether a given reaction could occur, with no consideration of the reaction rate. Recently, Chatterjee et al.^4^ demonstrated the experimental implementation of tethered molecular logic circuits on DNA origami tiles. As outlined in Fig. 1, we use the hairpin-based signal transmission wire from that work as our running example to demonstrate our computational approach. Using the same inter-hairpin distance and domain lengths as in that paper, we use a number of simple, parameterized biophysical models to estimate the ensemble of possible physical conformations of the structures, and use these to estimate the reaction rates between tethered molecular species via the local concentration approach. Taking account of domain lengths and angles between the domains, we first generate the structures and then we predict the probability that the two complementary domains will be colocated, given the biophysical constraints. Then we use this information to estimate the local concentration for the reaction system and compare it to a valued inferred from previous experimental work^4^. This work therefore develops a system that uses simple biophysical models to automatically derive estimates of this parameter via a “bottom-up” approach.

## Results

### Parameterized biophysical model

We made some assumptions to simplify our biophysical model. First, we model the structures under study as DNA domains connected by joints. Each domain is either double-stranded or single-stranded. We model double-stranded domains as rigid rods and single-stranded domains as freely-jointed chains. The domains are connected by a joint which may be infinitely flexible or may be constrained, depending on the type of domains that it connects. We assume that the length of the bonds between the complementary bases is zero. This keeps the biophysical model simple, although parameterizing the model with distinct distributions for the lengths of, and angles between, domains allows us to investigate a range of models using this overarching framework, as outlined below. The choice made for the distributions of the biophysical parameters affects the structures generated. We consider four different models, termed UU, UN, WU, and WN, which are summarized in Table 1 and outlined in more detail in the “Methods” section. The key distinctions are the distributions used to model the lengths of the single-stranded domains and the possible angles between adjacent double-stranded domains. Briefly, the UU and UN models draw single-stranded DNA (ssDNA) domain lengths from a uniform distribution, whereas the WU and WN models draw ssDNA domain lengths from a worm-like chain distribution. Similarly, the UU and WU models draw angles between double-stranded DNA (dsDNA) domains from a uniform distribution, whereas the UN and WN models draw angles between dsDNA domains from a distribution derived empirically from previously reported computational modeling work^4^.

**Table 1:** Summary of the different biophysical model parameterizations investigated in this work.

	UU model	UN model	WU model	WN model
dsDNA length	Fixed	Fixed	Fixed	Fixed
**ssDNA length**	**Uniform dist.**	**Uniform dist.**	**WLC dist.**	**WLC dist.**
ssDNA-ssDNA angle	Uniform dist.	Uniform dist.	Uniform dist.	Uniform dist.
ssDNA-dsDNA angle	Uniform dist.	Uniform dist.	Uniform dist.	Uniform dist.
**dsDNA-dsDNA angle**	**Uniform dist.**	**Nicked dist.**	**Uniform dist.**	**Nicked dist.**
Tether angle	Uniform dist.	Uniform dist.	Uniform dist.	Uniform dist.

All four models use identical distributions for dsDNA domain lengths, ssDNA-ssDNA domain angles, ssDNA-sdDNA domain angles and tether angles. They differ only in the choices made for ssDNA domain lengths and dsDNS-dsDNA domain angles, highlighted in boldface in the table. “WLC dist.” refers to the worm-like chain model of length distributions of jointed chains. “Nicked dist.” refers to a distribution of inter-domain angles at a nick “joint between two dsDNA domains linked only on one strand” that was previously reported^4^. All distributions are described in detail in the main text.

### Structure sampling to analyze a localized hairpin interaction

**Figure 2:**
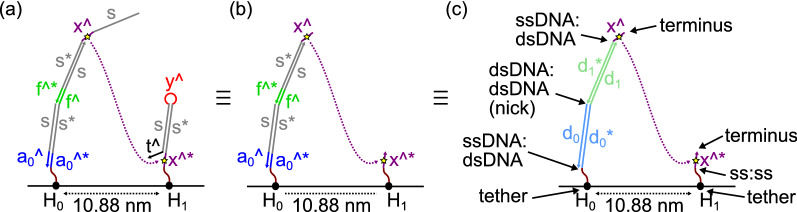
The specific tethered structures investigated in this paper, after Chatterjee et al.^4^ Small yellow stars indicated the reactive points defined for each structure. (**a**) The hairpins $H_0$ and $H_1$, with input and fuel molecules having bound to $H_0$ and opened it. (**b**) To simplify the analysis, we remove all domains further from the tether than the complementary ${x}^{\wedge}$ toeholds, producing these minimized structures. (**c**) Classification of the different kinds of junction between domains that occur in these structures. Each of the labeled junctions, plus the strand terminus, will be assigned random coordinates via our sampling algorithm. In addition, this image shows the condensing of multiple adjacent double-stranded domains of the same type into a single domain, which further simplifies the model.

We used the structure sampling approach (outlined in “Methods”) to analyze the intramolecular step in a hairpin-based signal transmission wire of the kind developed by Chatterjee et al.^4^ The whole reaction scheme is shown in Fig. 1 and the particular structures under investigation in this work are illustrated in Fig. 2. Structure $H_0$ represents an input hairpin that has been opened by an input strand via toehold-mediated strand displacement and subsequent binding to a freely diffusing fuel hairpin, and is composed of both single-stranded and double-stranded domains. Structure $H_1$ represents the first hairpin in a signal transmission wire, which is waiting to be opened via a localized interaction with structure $H_0$. Our goal is to determine the rate of interaction between these structures, which will interact initially via binding of the ${x}^{\wedge}$ toehold on $H_0$ to the complementary toehold ${{{x}^{\wedge *}} }$ on $H_1$. Therefore, we just need to determine the probability that these two domains are positioned close enough to each other to interact. To simplify the sampling process, we remove those domains that are further from the tether than the toehold in question, as these will not affect the position of the toehold in each case (see Fig. 2a,b). We also condense adjacent domains of the same type (either single-stranded domains or double-stranded domains that are connected on both strands, i.e., not separated by a nick) into a single domains whose length is the sum of the two condensed domains. Specifically, in this example, domains ${a_0}^{\wedge}$ and *s* are condensed into $d_0$ and domains ${f}^{\wedge}$ and *s* are condensed into $d_1$, as shown in Fig. 2c. This not only simplifies the model but also means that the only coordinates sampled are the tethers, the strand termini, and the joints at which the DNA would actually flex. Note, however, that we do not condense the ${x}^{\wedge}$ domain with the tether spacer on the $H_1$ structure as it takes part in the interaction of interest and therefore we need to sample coordinates for the two ends of this domain specifically.

**Figure 3:**
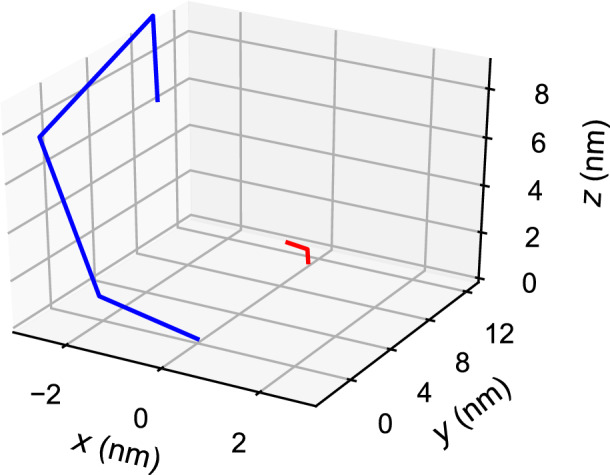
An example of a pair of sampled tethered structures. The $H_0$ structure is shown in blue and the $H_1$ structure is shown in red.

Using the procedure outlined above and presented in detail in the “Methods” section, we generated five datasets of randomly sampled structures for the $H_0$ and $H_1$ structures. Each individual sample consists of $({{x},{y},{z}})$-coordinates for each of the points labeled in Fig. 2c: tethers, junctions between contiguous domains, and strand termini. By convention, $H_0$ is tethered at $({{0},{0},{0}})$, and the directions of the axes are as specified in Fig. 1. We used an inter-hairpin distance of 10.88 nm, as this was the distance used by Chatterjee et al. to achieve localized signal transduction in their work^4^. The only exception to this is one dataset that uses a doubled inter-hairpin distance of 21.76 nm, which serves as a negative control dataset in which the rate of interaction between $H_0$ and $H_1$ should be negligible. An example of a pair of sampled structures is shown in Fig. 3. To compare the effects of changing between our different biophysical models on the generated structures, we generated structures for all four models (UU, UN, WU, and WN). To study the effects of sampling error on our results, we generated datasets of size $10^3$, $10^4$, $10^5$, and $10^6$ for each model.

To present aggregated information on the sampled structures, we define the *reactive point* of a structure as the specific coordinate that we will consider when calculating the probability of a particular interaction involving that structure. In this example, the DNA domains of interest are the ${x}^{\wedge}$ toehold on the $H_0$ structure and the complementary toehold ${{x}^{\wedge *}}$ on the $H_1$ structure, as these are exposed complementary domains that can undergo a hybridization reaction (that would lead to a subsequent toehold-mediated strand displacement reaction, although we do not consider that reaction in this paper). Therefore, the reactive points for the two structures under consideration here will be the *midpoints* of the two toehold domains ${x}^{\wedge}$ and ${{x}^{\wedge *}}$, which we calculate by finding the midpoint of a straight line between the coordinates sampled for the two ends of each of these toehold domains. To clarify this definition, the reactive points on the two structures used in this study are annotated as small yellow stars in Fig. 2.

**Figure 4:**
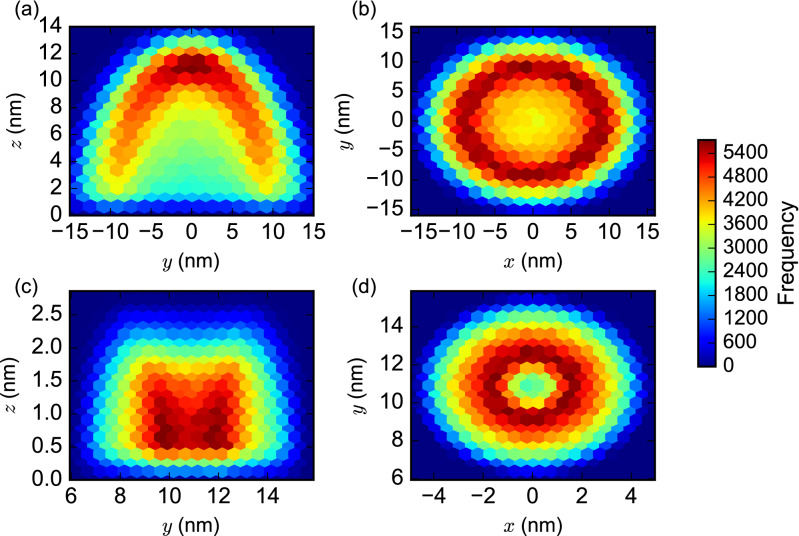
2-dimensional heat maps of the reactive point of $10^6$ samples of the $H_0$ and $H_1$ structures generated using the WN model. (**a**) $({{y},{z}})$ projection of reactive point of $H_0$ structures into the $x=0$ plane. (**b**) $({{x},{y}})$ projection of $H_0$ structures into the $z=0$ plane. (**c**) Similar $({{y},{z}})$ projection of $H_1$ structures. (**d**) Similar $({{x},{y}})$ projection of $H_1$ structures. See Fig. 1 for the directions of the *x*, *y*, and *z* axes.

A summary of $10^6$ sampled locations of the reactive points of structures $H_0$ and $H_1$ generated using the WN model are shown in Fig. 4. In each panel, the samples of the location of the reactive point are projected onto either the $({{y},{z}})$ or $({{x},{y}})$ plane. Fig. 4a and b show (the reactive point of) $H_0$ projected into the $x=0$ and $z=0$ planes, respectively. Similarly, Fig. 4c and d show (the reactive point of) $H_1$ projected into the $x=0$ and $z=0$ planes, respectively. The $H_0$ structures were generated with their tether at the origin, whereas the $H_1$ structures were generated with their tether at (0,10.88,0), thereby providing the 10.88 nm inter-tether distance outlined above. The $x=0$ projections may be thought of as “side views” of the spatial distribution of the reactive point in each case, and the $z=0$ projections may be thought of as “views from above”. Similar summaries of reactive point structure samples for the other biophysical models with $10^6$ samples are provided in Supplementary Figs. S1 (UU model), S2 (WU model), and S3 (UN model).

These sampling results exhibit some clear differences depending on which of our four biophysical models was chosen. The shorter structure, $H_1$, contains only single-stranded domains. Therefore, the choice between the uniform and nicked distributions for the angle between two dsDNA domains separated by a nick (i.e., UU/WU models versus UN/WN models) has no affect on the structures that are sampled. For the longer structure, $H_0$, however, this choice makes a significant difference. The structure samples generated by the UN/WN models show a well-defined “shell” of maximal probability for the location of the reactive point, $\thicksim$ 10 nm from the origin. These can be seen clearly in both the “top” and “side” views shown in Fig. 4. The structures generated by the UU/WU models, on the other hand, have reactive points clustered around the origin within a radius of around 4 nm. These differences are likely due to the fact that the nicked angle distribution produces $H_0$ structures in which neighboring nicked domains are more likely to be oriented in roughly the same direction (see Supplementary Fig. S24). Thus, the initial direction selected for the first domain adjacent to the tether is likely to be very important in determining the overall direction of the structure and thus the location of its endpoint. On the other hand, the uniform angle distribution will tend to produce far more structures in which the domains fold back on each other at extreme angles, creating the observed concentration of reactive points around the origin.

The distribution from which to sample the lengths of ssDNA domains (i.e., UU/UN models versus WU/WN models) does not significantly impact the overall shape of the distribution of sampled structures. It does seem to slightly impact its spread, for example, the WN model produces a slightly more compact distribution for $H_0$ than the UN model, however, this is reversed for $H_1$. The WU model seems to produce more spread-out distributions for both $H_0$ and $H_1$ than the UU model.

Based on the above considerations, we determined that the WN biophysical model seems to provide the most reasonable and realistic model of the structures under study. This is perhaps not a surprise given that it incorporates more detailed biophysical data than any of the other options. As we discuss below, there are also reasons to favor the WN model based on how it predicts the system of Chatterjee et al.^4^. Therefore, the analyses presented in the main text below all use the WN model as the starting point. We have, however, carried out all analyses on all four models of the system, and the results from those analyses are presented in the Supplementary Information for comparison.

### Local concentration results for the localized hairpin interaction

After generating the hairpin structures, we used these generated structure distributions to estimate the probability that the two reactive points on the two tethered strands would be colocated in 3D space at any given moment. We then used this probability to estimate the local concentration; the details of these calculations are presented in the “Methods” section below. Briefly, we consider any pair of structures whose reactive points are within a threshold distance of 2 nm (the justification for this value is presented in the Discussion below) to interact. Then, for each sampled $H_1$ structure, we calculate the fraction of the $H_0$ structures that put the reactive points within this threshold distance. We interpret this fraction as a probability, and from this we arrive at a local concentration *observed by* $H_1$ *at each point* by scaling by the volume enclosed by the threshold distance around the reactive point of $H_1$. We then average across all these values to produce a single value for the local concentration associated with this reaction. This can be interpreted as the equivalent concentration of one of the localized reactants as observed by the other, and allows us to scale kinetic rate constants for these interactions from their observed values for bimolecular interactions in bulk solution to produce equivalent rate constants for the localized interactions.

**Figure 5:**
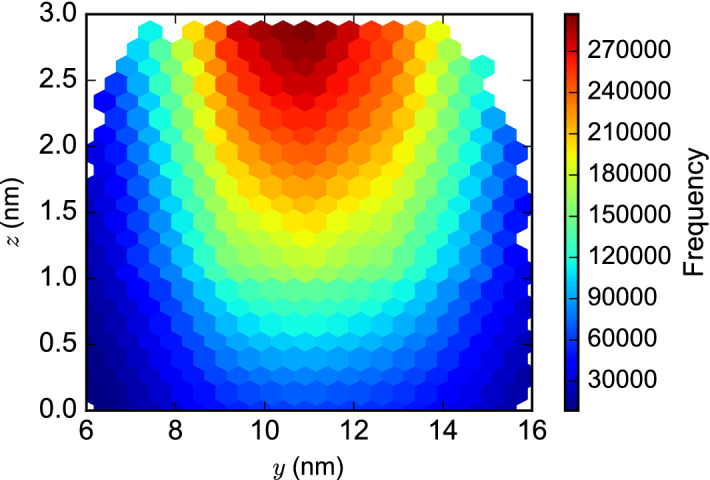
A (*y﻿*, *﻿z*) hexbin plot of local concentrations computed at sampled locations of the reactive point of the $H_1$ structure, for one dataset of $10^6$ sampled structures.

To decide whether two strands are in close enough proximity to interact, we use 2 nm as our default threshold size. This means that the sphere of potential interaction has diameter 4 nm. Given that we assume that the length per nucleotide in single-stranded DNA is 0.68 nm, this sphere diameter is $\thicksim$ 6 nucleotides, the same length as the toehold domains used by Chatterjee et al.^4^. We did study the effect of changing the size of the threshold for the WN model, and these data are presented in Supplementary Fig. S21. From that plot, we observe that with an increase in the threshold size, there is a minor decrease in the local concentration values; this effect is significantly smaller than the effects of other possible changes such as changing to a different model. As the threshold size increases, the volume of the region in which we search for the colocation of the reactive points of the two structures, increases as the cube of the radius. This volume is the denominator in the calculation used to convert colocation probabilities into localized concentrations (see “Methods”), and the volume seems to increase slightly faster than the associated probability, thereby causing a slight decrease in the resulting calculated overall value for the local concentration.

Figure 5 presents a heatmap of the estimated local concentration values observed at different sampled positions of the reactive point of the $H_1$ structure, using the WN model. The colored area shows the volume of overlap between $10^6$ sampled location distributions for the reactive points of the $H_0$ and $H_1$ structure, projected into the $x=0$ plane, as before. The striking feature of this plot is the region of maximal local concentration calculated at $y \approx {11} \, {\text{nm}}$, which is located almost directly above the tether position for the $H_1$ structure, located at $y = {10.88} \, {\text{nm}}$. As expected, this corresponds to the region of maximal overlap of the $H_0$ and $H_1$ structures as plotted in Fig. 4. The corresponding plot for the UN distribution is similar (Supplementary Fig. S18). These contrast with similar plots for the WU model (Supplementary Fig. S17) and the UU model (Supplementary Fig. S16), which show the largest local concentration observed in the region of overlap that is closest to the $H_0$ structure, at $y \approx {6}\, {\text{nm}}$. These plots may be thought of as identifying the region of space in which the interaction between the ${x}^{\wedge}$ toehold on $H_0$ and the complementary toehold ${{x}^{\wedge *}}$ on $H_1$ is most likely to occur. As we observed in the distributions of the reactive points, the choice between the uniform and nicked distributions for the angle between two dsDNA domains separated by a nick (i.e., UU/WU models versus UN/WN models) has the most impact on the resulting area. The difference in the distribution of the areas of highest local concentration is of practical interest, as outlined in the Discussion below.

To highlight the effects of sample size on the resulting distributions of local concentration, similar plots are presented in the Supplementary Information for each of our five generated datasets consisting of either $10^3$ samples (Supplementary Figs. S4–S7), $10^4$ samples (Supplementary Figs. S8–S11), $10^5$ samples (Supplementary Figs. S12–S15), or $10^6$ samples (Supplementary Figs. S16–S19). As expected, we observe greater similarity between the five replicate plots when the number of samples is greater; as the number of samples drops to $10^4$ or even $10^3$ we observe significant differences due to sampling error. Therefore, we must be careful to use a large enough number of samples to produce reliable estimates: hence we have used the values calculated from $10^6$ samples throughout, unless otherwise stated.

The overall calculated values for the local concentration are shown in Table 2, for the WN model with a threshold size of 2 nm. The calculated values are the averages across five different datasets containing $10^4$, $10^5$, and $10^6$ samples. The values computed for an inter-hairpin distance of 10.88 nm are $\thicksim$ ${132}\upmu \mathrm{M}$. This is within roughly a factor of two of the value inferred from experimental results by Chatterjee et al.^4^ which was $\thicksim$ ${60}\upmu \mathrm{M}$. This shows that our simple model can give results that are comparable to experimentally derived values. (A plot comparing these values to similar values calculated for the other three models is presented as Supplementary Fig. S20). To study the effect of the inter-hairpin distance on the local concentration, we carried out a similar computation for a double-spaced 21.76 nm inter-hairpin distance (see Table 2 and Supplementary Fig. S22). That data shows a local concentration of $\thicksim {24} \, \mathrm{nM}$, which is roughly 10,000 times lower than the value for the 10.88 nm spacing. This corresponds to data from previous work^4^ that used the 21.76 nm double-spaced hairpin as a negative control reaction that did not produce viable signal propagation. Therefore, the results from our calculations reproduce observed behavior of localized circuits both qualitatively and, within roughly a factor of two, quantitatively.

**Table 2:** Summary of local concentration values obtained for WN model for different sample sizes for inter-hairpin distances of 10.88 nm and 21.76 nm.

Sample size	Inter-hairpin distance (nm)	Local concentration (nM)	Unimolecular rate constant (${s^{-1}}$)
$10^{4}$	10.88	$131083.60 \pm 15659.65$	$\thicksim 655 \pm 78.29$
$10^{5}$	10.88	$130535.49 \pm 3088.38$	$\thicksim 652 \pm 15.44$
$10^{6}$	10.88	$132587.14 \pm 1183.33$	$\thicksim 662 \pm 5.91$
$10^{4}$	21.76	$44.49 \pm 20.5$	$0.222 \pm 0.102$
$10^{5}$	21.76	$21.34 \pm 10.43$	$0.106 \pm 0.052$
$10^{6}$	21.76	$24.25 \pm 3.02$	$0.121 \pm 0.0151$

A threshold distance of 2 nm between the reactive points was used to determine whether two particular structures may interact. Unimolecular rate constants were computed from multiplying the local concentration values by the bimolecular rate constant of the corresponding species if they were freely diffusing in solution. This bimolecular rate constant is the value $k_{\{0,6\}}=5 \times 10^{5} M^{-1} s^{-1}$ estimated by Zhang and Winfree^17,27^, which is chosen because the system reported by Chatterjee et al.^4^ employed a 6-nucleotide toehold on the invader with no distal toehold on the incumbent. Local concentration and unimolecular rate constant values are expressed as mean ± standard deviation of five sampled datasets of each size.

To investigate the effect of sampling on our final calculated values of the overall local concentration, Table 2 shows the calculated local concentration against the total number of structure samples for the WN model. Similar results for the other three model variants are presented in Supplementary Fig. S20. These results show that, even for just $10^4$ samples, the mean calculated local concentration is very similar to that obtained with significantly more samples ($10^5$ or $10^6$). In particular, $10^5$ samples seems to offer a good compromise between a good estimate of the final local concentration value and requiring less computation time to produce the ensemble of samples and analyze the results. In addition, as shown in Supplementary Fig. S20, our different models predict slightly different values for the local concentration. The UU, WU, and WN models produce local concentration values that are comparable ($\thicksim$ ${130}\upmu \mathrm{M}$), while the values from the UN model are somewhat higher ($\thicksim$ ${200}\upmu \mathrm{M}$). The UN model seems to produce a somewhat larger volume with high local concentration, closer to the $z=0$ plane of the DNA origami tile. This may be because the uniform angle distribution allows more flexibility at nicks, enabling more structures to be sampled nearer to the plane of the tile.

## Discussion

In this paper, we have studied the role that molecular geometry plays in the dynamics of the localized molecular circuits. Our results show that the orientation of structures generated in 3D space are dependent on the choice of the parameters made when we incorporated geometrical constraints into our biophysical model of the system. The local concentration values obtained from our WN model are $\thicksim$ ${130}\upmu \mathrm{M}$. Importantly, this is within the range of previously reported values. In particular, for the specific experimental system that we study here, Chatterjee et al.^4^ inferred a value of $\thicksim$ ${60}\upmu \mathrm{M}$ by Bayesian parameter fitting based on bulk fluorescence data. In addition, the value obtained for hairpins placed twice as far apart was significantly smaller ($\thicksim$ 20 nM), which agrees with the experimental result that a system containing double-spaced hairpins did not exhibit significant interactions^4^. In earlier work, Dalchau et al.^24^ had used the worm-like chain biopolymer model to determine the total length of both of the single stranded DNA and double stranded DNA in a similar hairpin system, and estimated a higher local concentration value of ${1000}\upmu \mathrm{M}$. The results obtained from our model are therefore comparable to those estimated in previous work. Localized reaction rates, and hence local concentration values, are notoriously difficult to measure accurately due to the high speed of the interactions. Therefore, computational modeling is expected to remain an important aspect of modeling these systems and our work contributes to this effort. These local concentrations can then be multiplied by rate constants derived for corresponding solution-phase reactions^12,17^ to produce unimolecular rate constants for kinetic modeling of the localized reactions.

We have presented four models, which we call UU, WU, UN and WN. Each of these incorporates some biophysical assumptions, and we used all four to generate structures subject to the geometric constraints imposed by the biophysical assumptions. Among these models, the WN model appears to be a fairly realistic model that is also consistent with previous experimental work, as outlined below. In our example system, in the WN model we observe the greatest overlap between two structures. In the WN model, the use of the worm-like chain distribution for the lengths of single-stranded domains biases toward elongation of the DNA strands compared to models in which the lengths are uniformly distributed. The WN model’s use of the “nicked” distribution for the angles between double-stranded domains separated by a nick, which was derived from the molecular dynamics simulation of oxDNA and in which most of the angles lie between roughly $20^{\circ }$ and $30^{\circ }$, produces structures that are less inclined to fold back on themselves than those models that choose such angles from a uniform distribution. The interplay of these two parameters means that the generated structures, as shown in Fig. 4, line up well with the experimentally chosen distance of 10.88 nm between neighboring hairpins in this localized reaction system^4^. This lends credence to our choice of this model as a reasonable one for subsequently determining the corresponding local concentration.

The experimental work that served as the basis for our study^4^ used DNA origami as a tethered surface. In practice, electrostatic repulsion between the DNA surface and the tethered DNA circuit components could impact the distribution of the structures. In this work, we modeled the initial tethered angle as a uniform distribution. However, this could be easily changed to a different, non-uniform distribution that is biased against angles that produce directionality vectors for the initial domain that are parallel, or almost parallel, to the surface. This would provide a simple mechanism to model some aspects of this repulsion effect. In addition, another simple mechanism to incorporate broader effects of electrostatic repulsion would be to modify our rejection sampling procedure so that, rather than eliminating all samples with $z < 0$, we could reject all samples with $z < \epsilon$, for some positive constant $\epsilon$, thereby preventing any part of the structure from coming too close to the surface. Simulation results demonstrating the effect of this change on the overall local concentration value are presented in Supplementary Fig. S25, showing that adding some element of repulsion does decrease the estimated local concentration value, making it closer to the inferred value from prior work^4^.

The estimations of local concentrations for localized interactions from our work could be used as part of an automated system to enumerate localized reactions and generate kinetic models of localized DNA strand displacement systems, building on previous work to model and simulate such systems^4,21^. This would simplify the expansion of our work to model other localized reaction systems^3,28^, including an interesting, recently published system for localized signaling induced by conformational changes in the underlying DNA origami nanostructure^29^. In addition, our approach could be used to model other kinds of localized system such as DNA-based molecular walkers^30–32^. This will also require further extensions of this work to model more larger structures, in particular, those in which the conformational ensemble is constrained by multiple tethers rather than just one. In addition, incorporating more detailed experimental data should help us to parameterize our biophysical model more realistically, enabling more accurate estimates of the localized reaction rates. This would help in building more accurate kinetic models of these localized systems. However, we expect that direct experimental validation of our models may be challenging because these reactions are very fast and therefore hard to observe directly.

## Methods

In this section, we explain in detail the procedure which we used to generate five datasets of sampled structures for the $H_0$ and $H_1$ structures and use these sampled structures to compute the local concentration. We have proposed four different models (UU, UN, WU, WN) which incorporate different geometrical constraints. First, we describe the parameters that distinguish these models. We then describe algorithm for generating populations of distinct structures given these parameters. Finally, we describe the method used to compute the local concentration.

### Domain length distributions

Each double-stranded domain is assumed to be of fixed length, calculated by multiplying the number of nucleotides in the domain sequence by the length per nucleotide in dsDNA, which we take to be 0.34 nm as previously reported^24^. As outlined in Table 1, the length of each single-stranded domain is calculated depending on the model in question. In the UU and UN models, the length of each domain is sampled from a uniform distribution over the interval [0, *m*], where *m* is the maximum possible length in nanometers, calculated by multiplying the number of nucleotides in the domain sequence by the length per nucleotide in ssDNA, which we take to be 0.68 nm as previously reported^24^. Here we effectively assume that ssDNA is infinitely flexible. In the WU and WN models, the degree of flexibility of the internucleotide linkage is modeled more reliably using the distribution derived from a “worm-like chain” model, which constrains the angle between neighboring nucleotides. A plot of this distribution is presented as Supplementary Fig. S23. We calculate the maximum possible length *L* of the ssDNA domain as before; we then sample domain lengths from the probability density function for a worm-like chain of length *L* and persistence length *s*, which is defined^33^ as:

$$\begin{aligned} p(R, s, L) = \frac{1}{L} \frac{4 \pi A r^2}{(1-r^2)^{9/2}} \exp {\left( -\frac{3t}{4(1-r^2)}\right) } \end{aligned}$$

where *R* is the length to which the worm like chain is extended within a range *dR*, with

$$\begin{aligned} A = \frac{4(3t/4)^{3/2} \exp (3t/4)}{\pi ^ {3/2} \left( 4 + \frac{12}{3t/4} + \frac{15}{(3t/4)^2 }\right) } \end{aligned}$$

where $t = L/s$ and $r = R/L$. We use $s = {2} \, nm$ for the persistence length of ssDNA^24^.

### Angle distribution between domains

In addition to sampling domain lengths, we must also sample the angles of the “joints” between those domains to create a full structure for each tethered component under consideration. The distribution from which we sample the relative angle in each case depends on the particular model parameterization and on the types of domain (double-stranded or single-stranded) on either side of the joint whose angle is being sampled. In all models, we assume that the angles between two ssDNA domains, and between an ssDNA domain and a dsDNA domain, are sampled to produce directionality unit vectors that are uniformly distributed in 3D space. However, to do this, we cannot just pick angles directly from the uniform distribution, as points picked this way will be clustered non-uniformly: this is effectively the polar angle in a spherical polar coordinate system, and if chosen uniformly from the interval $[{0},{2\pi })$, these angles would cluster around the poles. Therefore, to generate uniformly distributed polar angles we must scale as follows: we sample a value *x* from the uniform distribution over $({0},{1})$ and calculate the resulting angle^34^ as $\cos ^{-1}({2x-1})$. The initial orientation of a domain that is directly attached to a tether is sampled similarly, with the exception that we only sample from the hemisphere that is “above” the tile surface.

Junctions between two neighboring double-stranded domains that are joined via only one of the two strands are called *nicks*. In the UU and WU models, angles at nicks are also sampled from a uniform distribution, as outlined above. In the UN and WN models, these angles are sampled from a non-uniform distribution that more accurately represents the behavior of nicks, in which base-stacking between the ends of the two neighboring duplexes may be expected to favor configurations in which the two duplexes are pointing in similar directions. We call this the “nicked” angle distribution, and this derives from computational modeling carried out by Chatterjee et al.^4^ In that paper, the oxDNA tool^20^ was used to compute an estimate of this particular distribution. In this work, we sample from a histogram generated using the raw data calculated for that model in that paper, which we present as Supplementary Fig. S24.

### Structure sampling

**Figure 6:**
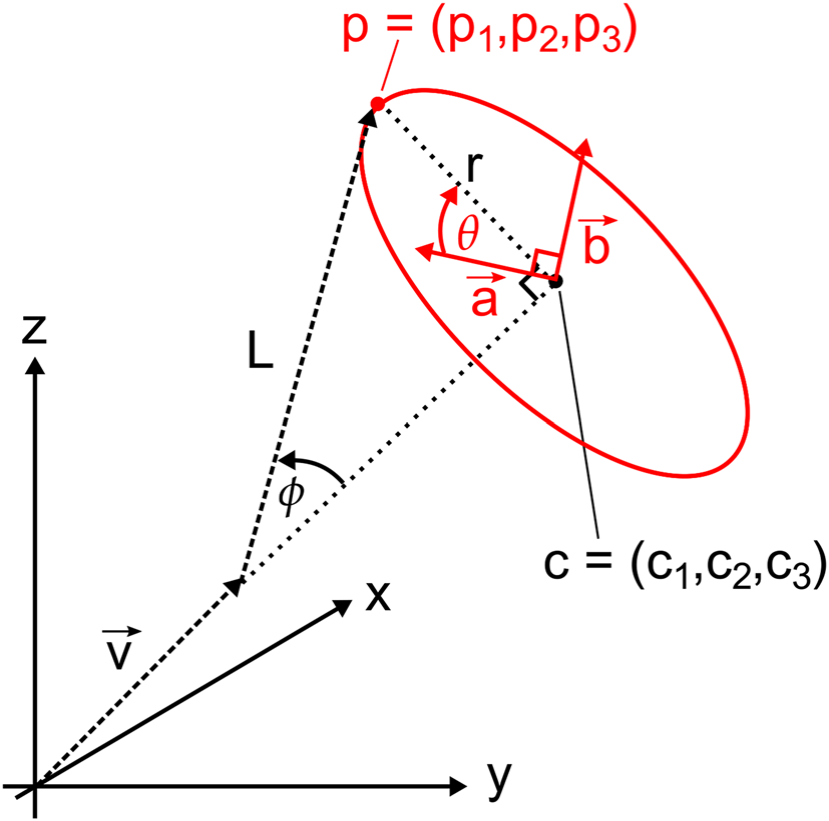
Vector diagram of the method used to generate structures. Briefly, we find the center *c* of a circle (shown in red) of possible locations from the previously generated parts of the structure along with the newly sampled length *L* and deviation angle $\phi$ for the current domain. We then obtain orthogonal unit vectors $\overrightarrow{a}$ and $\overrightarrow{b}$ in the plane of the circle and construct the parametric equation of the circle in 3D space in terms of *c*, $\overrightarrow{a}$, and $\overrightarrow{b}$. Finally, we randomly generate another angle $\theta$ to pick a point on that circle which we take as the sampled location of the distal end of the current domain.

Before sampling the structures, we first condense neighboring domains in the structure, as outlined in Fig. 2c. This ensures that the joints for which we sample angles are either at double-stranded nicks, or between single-stranded and double-stranded domains, or at either end of a single-stranded toehold domain that is involved in the particular interaction under study. Then, we use a simple algorithm to generate structures, as summarized in Algorithm 1, which describes the steps used in generating both the $H_0$ and $H_1$ structures. As both structures under study are linear, the inputs can be represented as a sequence of domains that are defined by their length in nucleotides and their type (single-stranded or double-stranded). The initial angle of the domain directly tethered to the tile surface is sampled as outlined above. From this, a unit vector is calculated that represents the direction from the end of that domain closest to the tether to the end of that domain furthest from the tether. The length of the domain is sampled as outlined above. For the directions of subsequent domains, we generate an angle that represents how much the next domain deviates from the directionality vector of the previous domain and use this to calculate the next directionality vector, as follows.

We first set up the 3D parametric equation of the circle which represents the rim of the cone of all points in 3D space that deviate by angle $\phi$ from the previous directionality vector $\overrightarrow{v}$, as shown by the red circle in Fig. 6. Given the previous domain’s unit vector $\overrightarrow{v} = ({{v_1},{v_2},{v_3}})$, we orient $\overrightarrow{v}$ to start at the origin. Thus, the “far end” of $\overrightarrow{v}$ is at $({{v_1},{v_2},{v_3}})$. Then, since the sampled angle is $\phi$, the centre of the red circle is $c = ({{c_1},{c_2},{c_3}})$, where:

$$\begin{aligned} c_1&= v_1 + ({v_1\cos ({\phi })}) \\ c_2&= v_2 + ({v_2\cos ({\phi })}) \\ c_3&= v_3 + ({v_3\cos ({\phi })}) \end{aligned}$$

Now, to set up the parametric equations of the circle we must find two unit vectors $\overrightarrow{a}$ and $\overrightarrow{b}$ which are perpendicular to each other and to $\overrightarrow{v}$. This means that they are in the plane of the circle, and are thus suitable to be used as orthonormal basis vectors for the parametric equations of the circle. To do this, we find a suitable vector for $\overrightarrow{a}$, and then take cross product of that vector with $\overrightarrow{v}$ to obtain $\overrightarrow{b}$. We observe that $\overrightarrow{a}$ must be perpendicular to $\overrightarrow{v}$, i.e., the inner product $\overrightarrow{a}\cdot \overrightarrow{v}$ must equal zero. This gives us:

$$\begin{aligned} ({a_1 \cdot v_1}) + ({a_2 \cdot v_2}) + ({a_3 \cdot v_3})&= 0. \end{aligned}$$

We note that at least one element of $\overrightarrow{v}$ must be non-zero, as the unit vector cannot have length zero. Without loss of generality, here we let $v_i$ stand for a non-zero element and let $v_j$ and $v_k$ stand for the other two elements of $\overrightarrow{v}$. Then, again without loss of generality, we fix the corresponding elements of $\overrightarrow{a}$ such that $a_j = a_k = 1$ and solve for $a_i$ based on the following, which is derived from the above equation for the inner product:

$$\begin{aligned} a_i&= \frac{-a_j\cdot v_j - a_k\cdot v_k}{v_i} \end{aligned}$$

By assumption, this equation will not involve division by zero since we picked $v_i$ to be a non-zero component of $\overrightarrow{v}$. We normalize $\overrightarrow{a} = ({{a_1},{a_2},{a_3}})$ to be a unit vector, then compute $\overrightarrow{b} = \overrightarrow{a} \times \overrightarrow{v}$. Since $\overrightarrow{a}$ and $\overrightarrow{v}$ are perpendicular unit vectors, $\overrightarrow{b}$ will also be a unit vector, which we write as $\overrightarrow{b} = ({{b_1},{b_2},{b_3}})$. The parametric equation of the red circle that represents the rim of the cone in Fig. 6 is then given by:

$$\begin{aligned} x({\theta })&= c_1 + ({a_1\, r \cos ({\theta })}) + ({b_1\, r \sin ({\theta })}) \\ y({\theta })&= c_2 + ({a_2\, r \cos ({\theta })}) + ({b_2\, r \sin ({\theta })}) \\ z({\theta })&= c_3 + ({a_3\, r \cos ({\theta })}) + ({b_3\, r \sin ({\theta })}) \end{aligned}$$

where $r = \sin ({\phi })$ is the radius of the circle. We then just need to sample a value for $\theta$ from the uniform distribution between 0 and $2\pi$ radians: this is essentially the azimuthal angle from spherical polar coordinates. We evaluate the parametric equations above for that value of $\theta$ to produce a uniformly-sampled point $p = ({{p_1},{p_2},{p_3}})$ on the circle. Finally, we obtain the next directionality unit vector $\overrightarrow{u} = ({{u_1},{u_2},{u_3}})$ from

$$\begin{aligned} u_1&= p_1 - v_1 \\ u_2&= p_2 - v_2 \\ u_3&= p_3 - v_3 \end{aligned}$$

and normalize $\overrightarrow{u}$ into the directionality unit vector for the next domain. We repeat this process until lengths and directionality unit vectors have been generated for all domains in the structure. (Note that the *coordinates* of all joints between domains can be reconstructed from the tether coordinates plus the lengths and unit vectors of all of the domains.)

In addition, while generating the structures we need to impose the constraint that no part of the structure should be “below” the underlying tile surface. In other words, we require that $z > 0$ for all generated $({{x},{y},{z}})$ coordinates in the structures. We could accomplish this in two ways. One way would be to compute the range of angles at each step of generating individual structures that would give rise to coordinates with $z > 0$ and only generate angles within that set. The second way would be to reject the structure when a coordinate with $z \le 0$ is generated and redo the sampling. The latter “rejection sampling” approach is far simpler from an algorithmic standpoint. We therefore adopt this approach in our structure sampling algorithm (Algorithm 1), with the exception that we only sample initial angles of domains directly adjacent to a tether from the hemisphere with positive *z*-coordinates. We estimated the fraction of the structure that we have to discard while sampling structures: these values are reported in Supplementary Table S1. For each valid structure generated, we estimate that an average of 4 structures must be discarded for the UU model, 3 structures for the UN model, 6 structures for the WU model, and 5 structures to be discarded for the WN model. This slows down the sampling process but is not prohibitive.

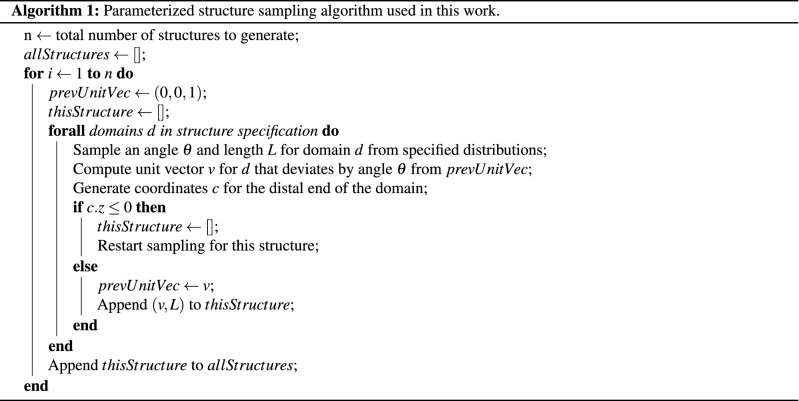



### Estimating local concentrations

Inspired by previous work that used the “local” or “effective” concentration approach to estimate the rate of reactions influenced by molecular geometry^25^, here we use a similar approach to estimate reaction rates. Thus, a key aspect of our method is the calculation of estimated local concentration. Then, to get the overall rate of the reaction, one could simply multiply this estimated local concentration with the rate constant of the corresponding solution-phase bimolecular interaction, e.g., those rate constants for toehold-mediated strand displacement reactions previously estimated by Zhang and Winfree^17^.

Based on our biophysical models, we produced ensembles of sampled tethered structures as outlined above. We now describe how we used these to produce an estimate of the corresponding local concentration for each model. The formula that we used to calculate local concentrations, or the effective concentration that one of the two toeholds may see of the other is:

$$\begin{aligned} C = \frac{P}{V} \end{aligned}$$

where *C* is in $\mathrm{nm}^3$, because our unit of length is the nanometer (see Supporting Information from Genot et al.^25^). Here, *P* is the probability that the reactive points of the two tethered structures $H_0$ and $H_1$ would colocate in 3D space and *V* is the reactive volume in $\mathrm{nm}^3$ or the volume where two reactive points have to colocate to be considered reacting. In most of our calculations, the reactive volume was a sphere of 4 nm diameter centered on the midpoint of one of the reactive toeholds, although we have also studied the effect of changing the size of this volume (see Supplementary Fig. S21). Our justification for using the 4 nm diameter sphere is that this length is roughly the same as 6 nucleotides, which is the length of the toehold domains used by Chatterjee et al.^4^ In any case, we know the volume of this sphere, and it just remains to calculate the probability of the two “reactive points” (midpoints of the two complementary toeholds) colocating within this volume.

To calculate the probability that the reactive point of the hairpin $H_1$ structure colocates with the reactive point of the another hairpin $H_0$ (or vice versa), we need to determine how many pairs of reactive points from $H_0$ and $H_1$ fall within the specified threshold distance. A naïve approach to this approach would be to calculate the distance between each of the sampled reactive points for $H_1$ and each of the reactive points for $H_0$. However, to speed up the process we use a binning technique. First, we categorize the individual samples into bins: cubes whose side length is the the same as our threshold distance for determining colocation of the samples. Therefore, in order to check which sampled locations for the reactive point of $H_0$ are within the threshold distance of a particular sampled location for the reactive point of $H_1$, we need only consider those sampled locations for $H_0$ that are either in the same bin as the point for $H_1$ or in one of the adjacent bins. Since the bins are cubes in 3D space, this means that we only need to search 27 bins, significantly reducing the search time. We then check the distance between each possible pair of sampled locations for the reactive points of $H_1$ and $H_0$ within those 27 bins to see if they are separated by a distance less than equal to the threshold distance. If so, they are counted as a reactive pair.

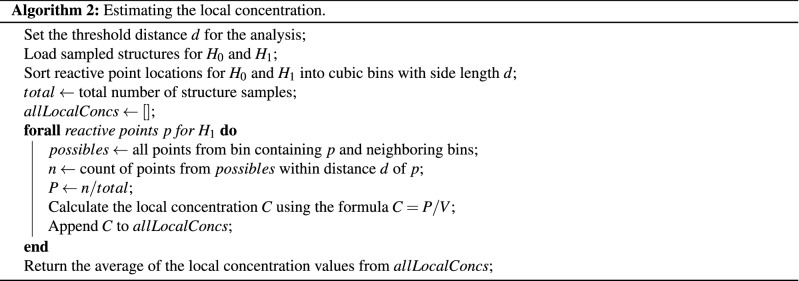



Algorithm 2 presents pseudocode for our algorithm to estimate the local concentration. First, a threshold distance is set which serves as the maximum distance a hairpin $H_1$ reactive point can be from a hairpin $H_0$ reactive point, for the two to be considered colocating in space. Then we read in every sampled reactive point from the sampled structures. We put these sampled points in bins based on the set bin width (threshold distance). For every sampled point from structure $H_1$, we look into the same bin and neighboring bins for the number of reactive point from structure $H_0$ that are within the threshold distance. For a given sampled point from $H_1$, the probability of any structure from $H_0$ being colocated with the $H_1$ point is just the count of data points within the threshold divided by the total number of points in the dataset. (Note that the choice of calculating the local concentration of $H_0$ as viewed from $H_1$ is arbitrary: we could have just as well done the calculation from the opposite perspective). We compute this probability for each point in the sampled dataset, and convert each to a local concentration using the above equation. We compute the local concentration of $H_0$ observed from every sampled location of $H_1$. Then, finally, we average these values to obtain an overall estimate of the local concentration value for the particular interaction under study.

Given that our unit of length in this work is the nanometer, this calculation produces a concentration value in particles/$\mathrm{nm}^3$, as outlined above. Our final task is therefore to convert this into a molar concentration. To do this, we multiply the concentration in particles/$\mathrm{nm}^3$ produced by the above equation by the following scale factor:

$$\begin{aligned} \frac{{1}\mathrm{mol}}{{6.022 \times 10^{23}}\mathrm{particles}} \cdot \frac{{1 \times 10^{27}\mathrm{nm}^3}}{{1}\mathrm{m}^3} \cdot \frac{{1}\mathrm{m}^3}{{1000}\mathrm{L}} \end{aligned}$$

This scaling factor produces a concentration in mol/L, i.e., in molar (M) concentration units. We can then straightforwardly scale this to other, more convenient, concentration units, such as $\mu \mathrm{M}$ or nM, as required.

## Supplementary Information

Supplementary Information.
