# Supplementary material for: Structure sampling for computational estimation of localized DNA interaction rates
Source: Sci Rep. 2021 Jun 16;11:12730. doi: 10.1038/s41598-021-92145-8 (PMC8209221; doi:10.1038/s41598-021-92145-8)
Supplement: Supplementary file 1 — Supplementary Information. [file 41598_2021_92145_MOESM1_ESM.pdf]

Structure sampling for computational estimation of localized DNA interaction rates
SUPPLEMENTARY INFORMATION

Sarika Kumar[1], Julian M. Weisburd[1], and Matthew R. Lakin[1,2,3]

[1]Department of Computer Science, University of New Mexico, Albuquerque, NM 87131, USA
[2]Department of Chemical & Biological Engineering, University of New Mexico, Albuquerque, NM 87131, USA
[3]Center for Biomedical Engineering, University of New Mexico, Albuquerque, NM 87131, USA

April 6, 2021

S1 Summaries of sampled structures for different biophysical models

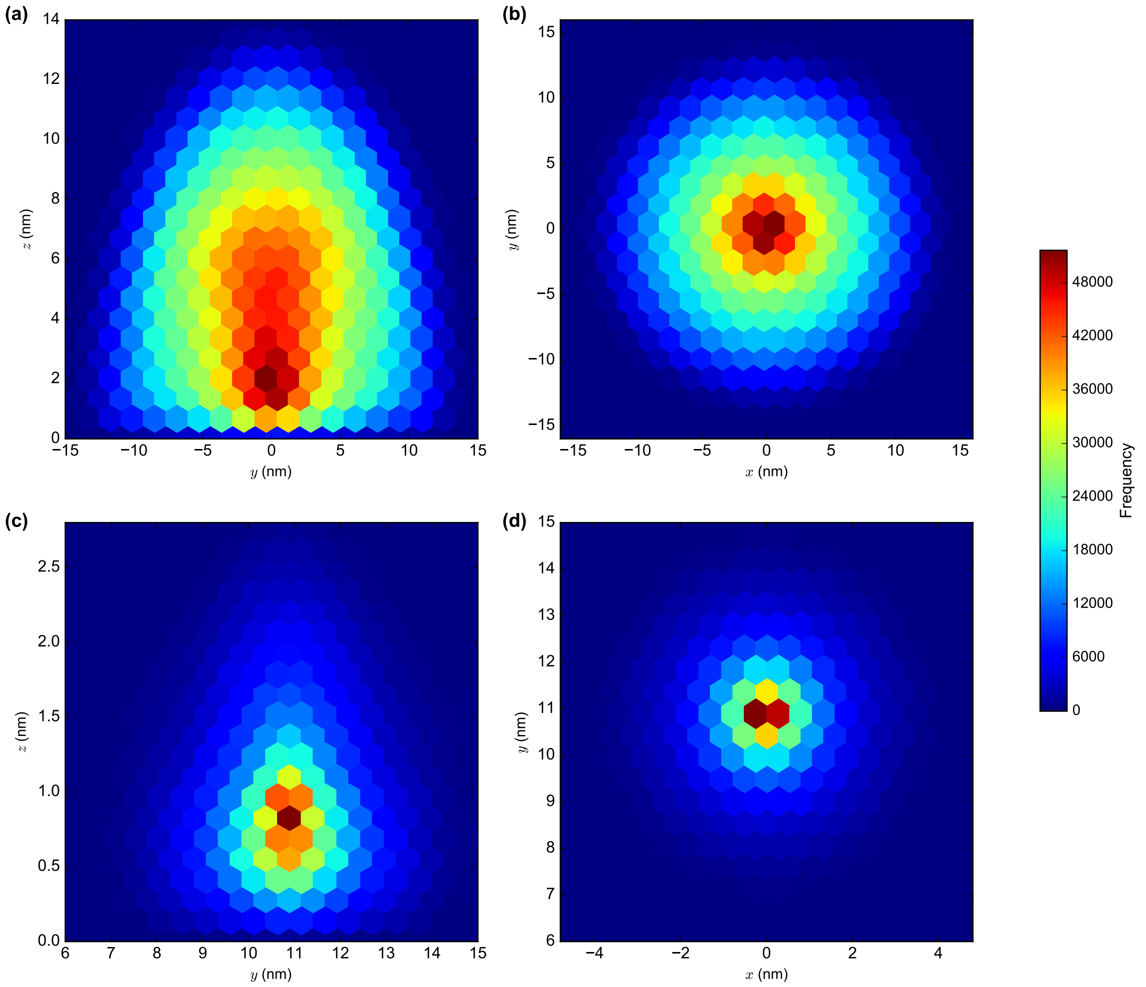

Figure S1: UU model structure sampling with sample size 10^6. **(a)** (y, z) projection of H_0 reactive point locations. **(b)** (x, y) projection of H_0 reactive point locations. **(c)** (y, z) projection of H_1 reactive point locations. **(d)** (x, y) projection of H_1 reactive point locations.

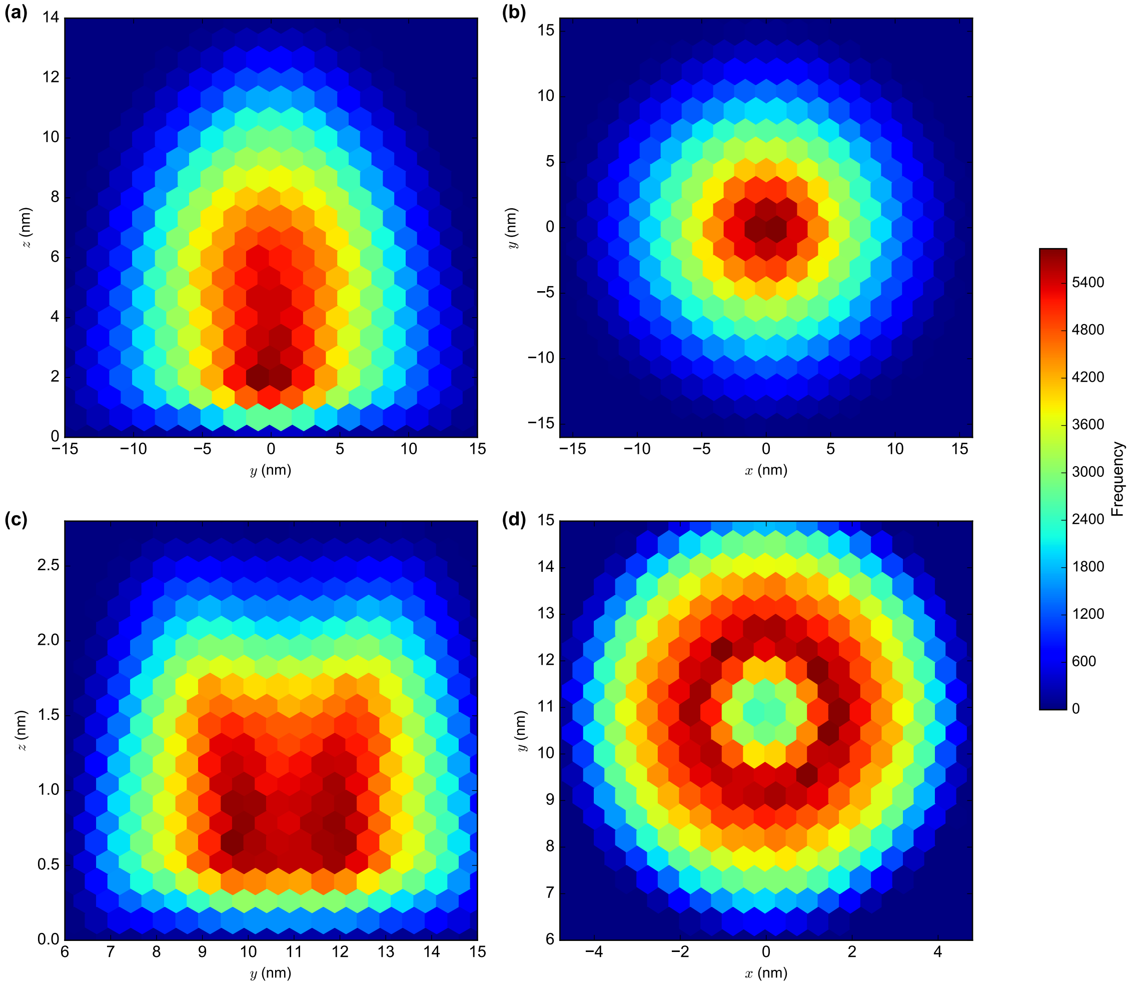

Figure S2: WU model structure sampling with sample size 10^6. **(a)** (y, z) projection of H_0 reactive point locations. **(b)** (x, y) projection of H_0 reactive point locations. **(c)** (y, z) projection of H_1 reactive point locations. **(d)** (x, y) projection of H_1 reactive point locations.

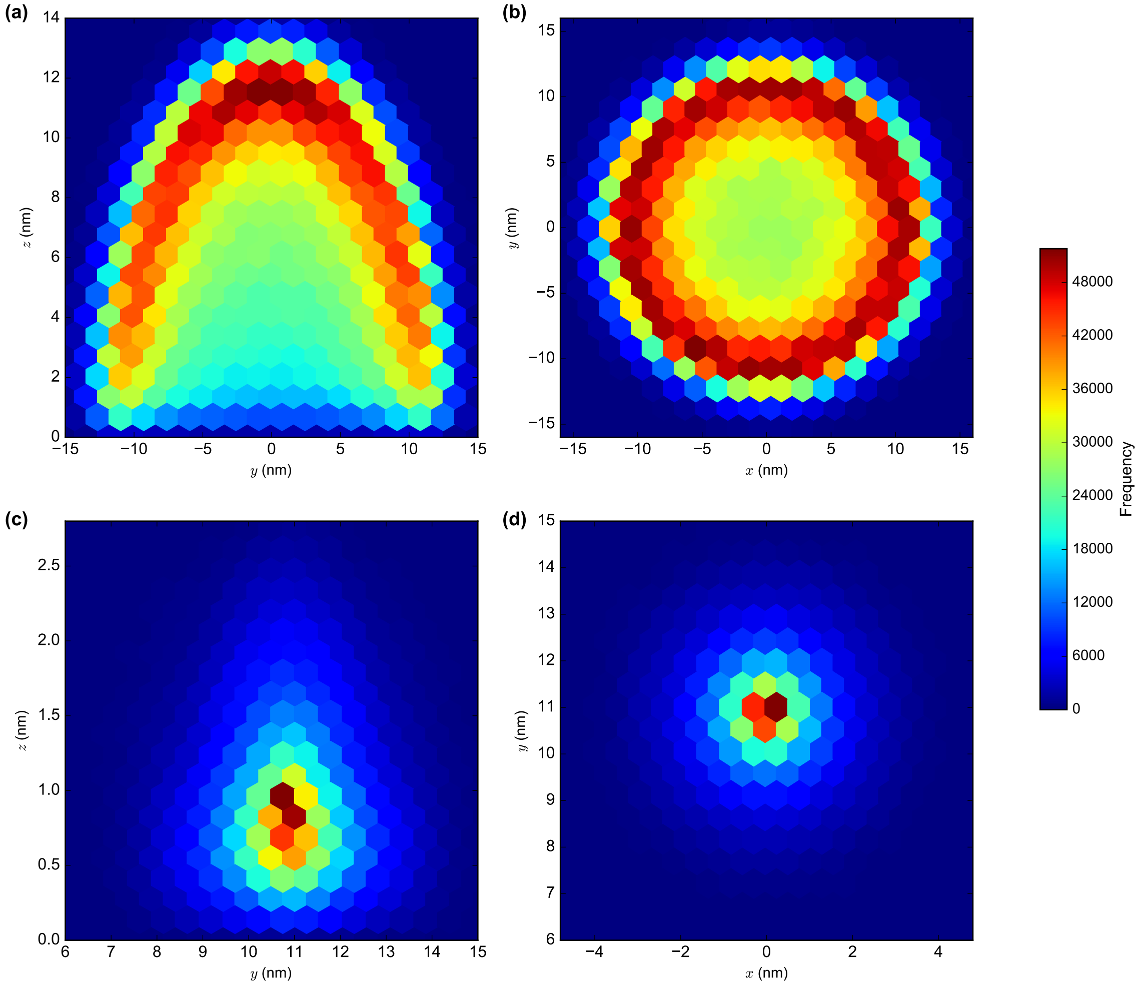

Figure S3: UN model structure sampling with sample size 10^6. **(a)** (y, z) projection of H_0 reactive point locations. **(b)** (x, y) projection of H_0 reactive point locations. **(c)** (y, z) projection of H_1 reactive point locations. **(d)** (x, y) projection of H_1 reactive point locations.

S2 Local concentration distribution for all models with 10^3 samples

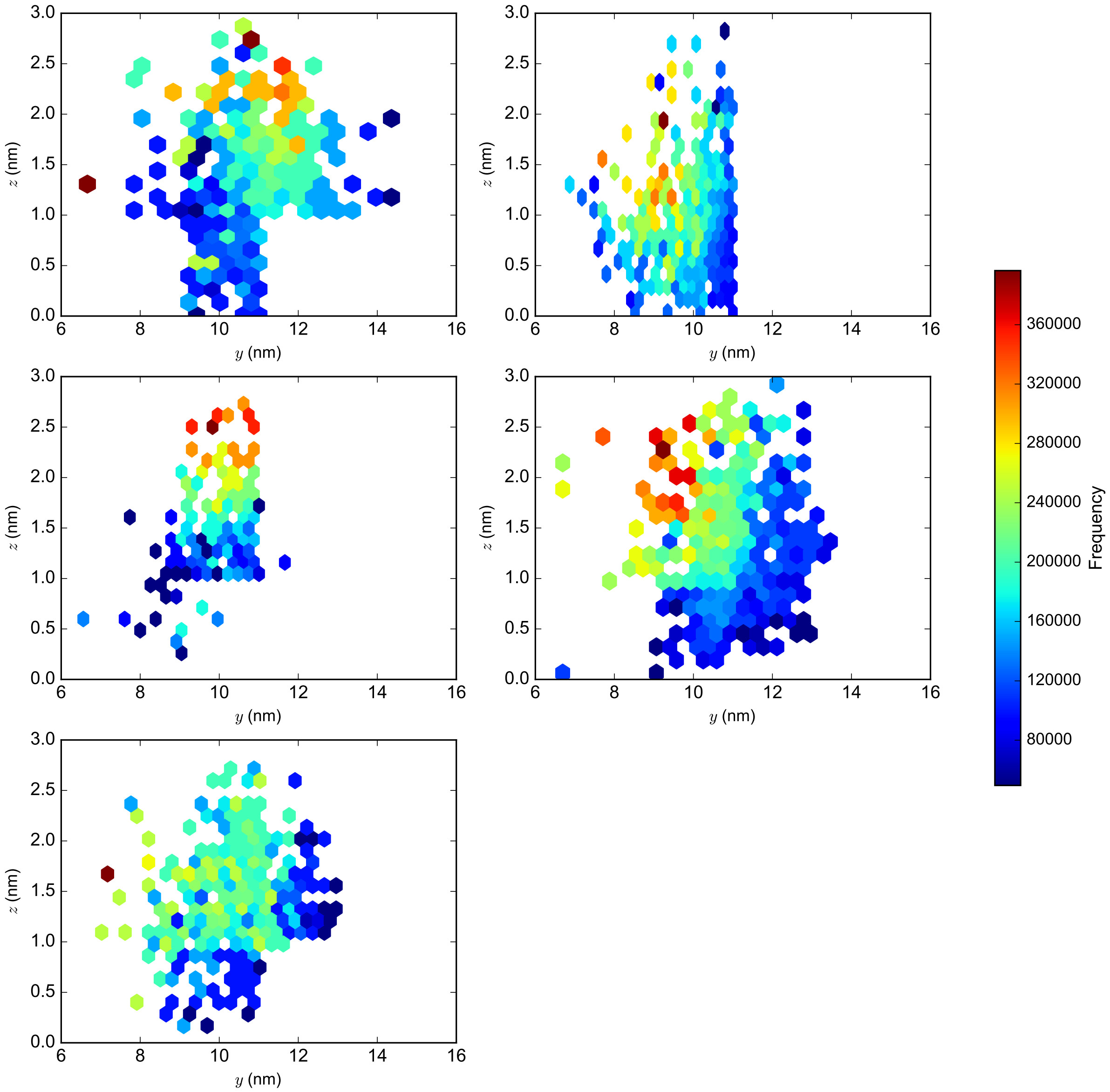

Figure S4: Spatial distribution of calculated local concentrations for five different datasets sampled using the UU model, each with a sample size of 10^3.

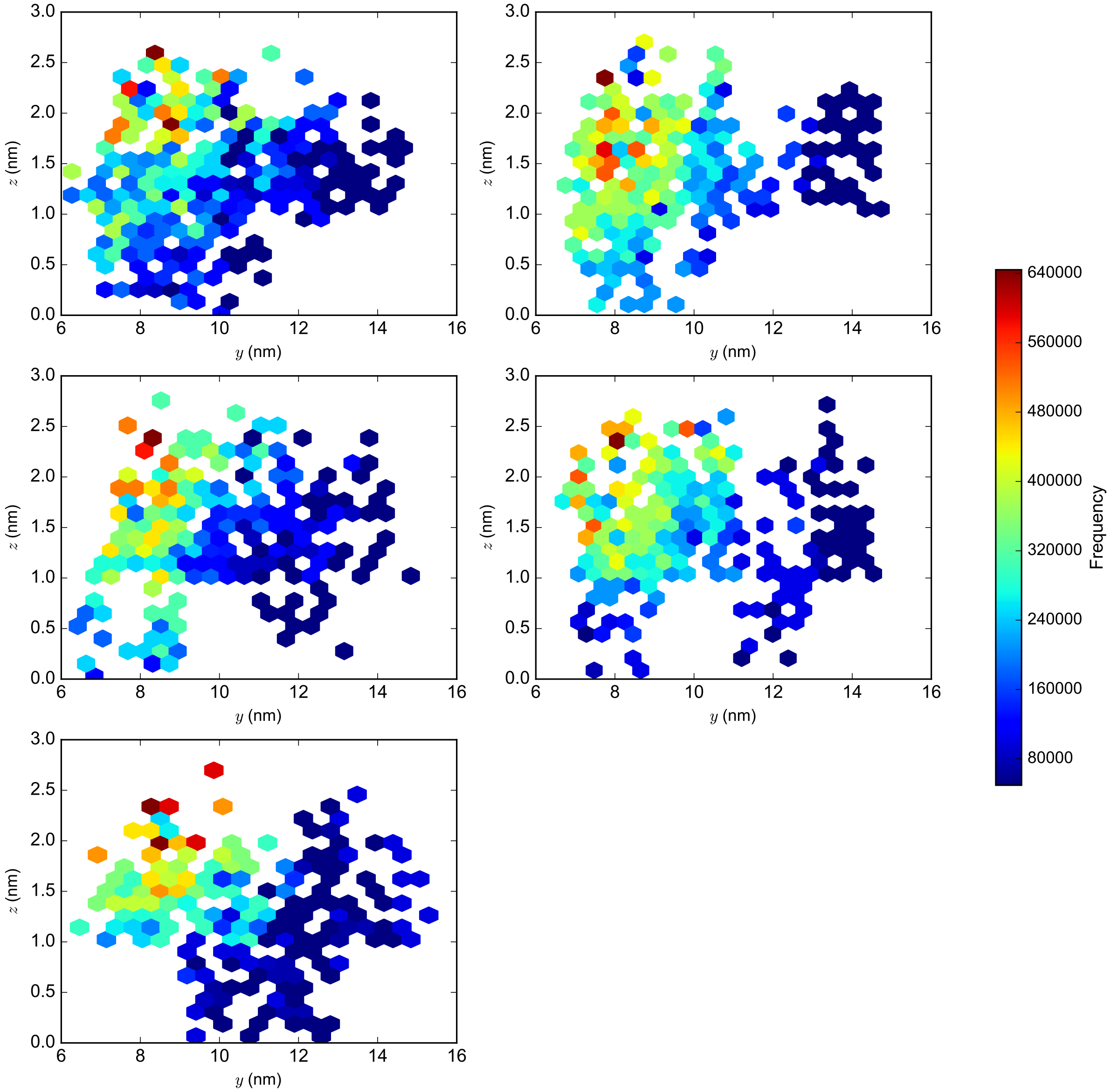

Figure S5: Spatial distribution of calculated local concentrations for five different datasets sampled using the WU model, each with a sample size of 10^3.

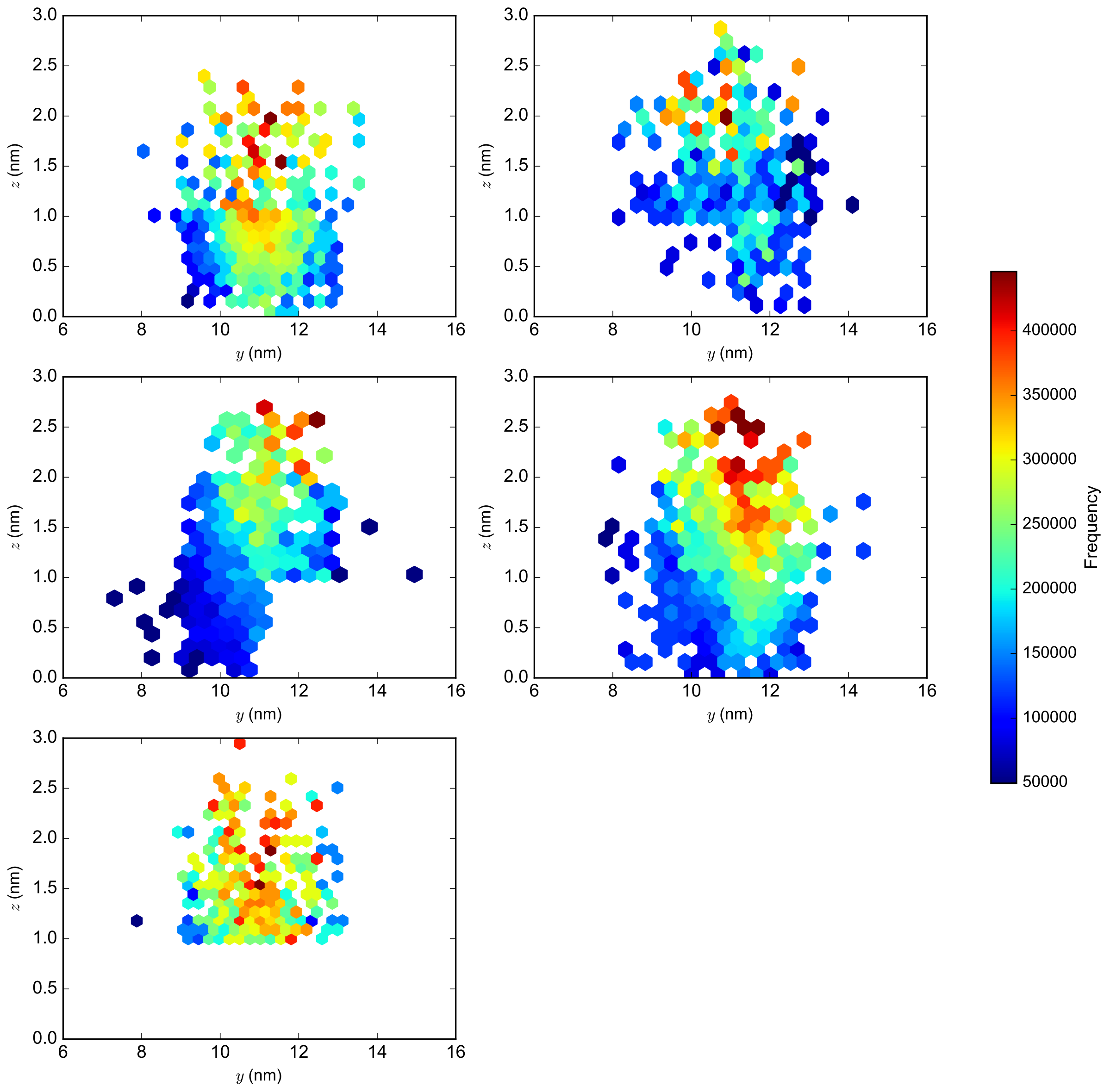

Figure S6: Spatial distribution of calculated local concentrations for five different datasets sampled using the UN model, each with a sample size of 10^3.

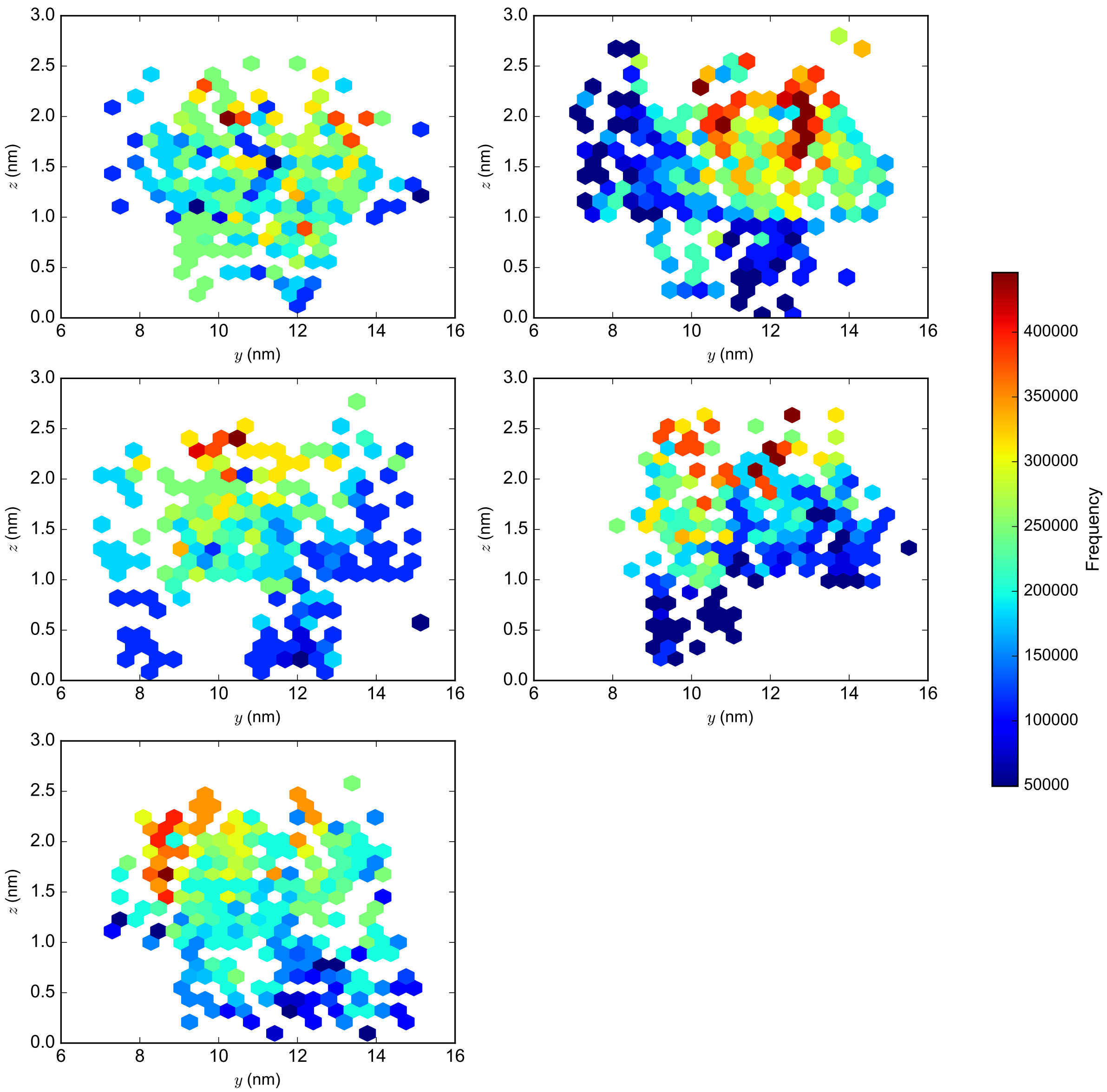

Figure S7: Spatial distribution of calculated local concentrations for five different datasets sampled using the WN model, each with a sample size of 10^3.

S3 Local concentration distribution for all models with 10^4 samples

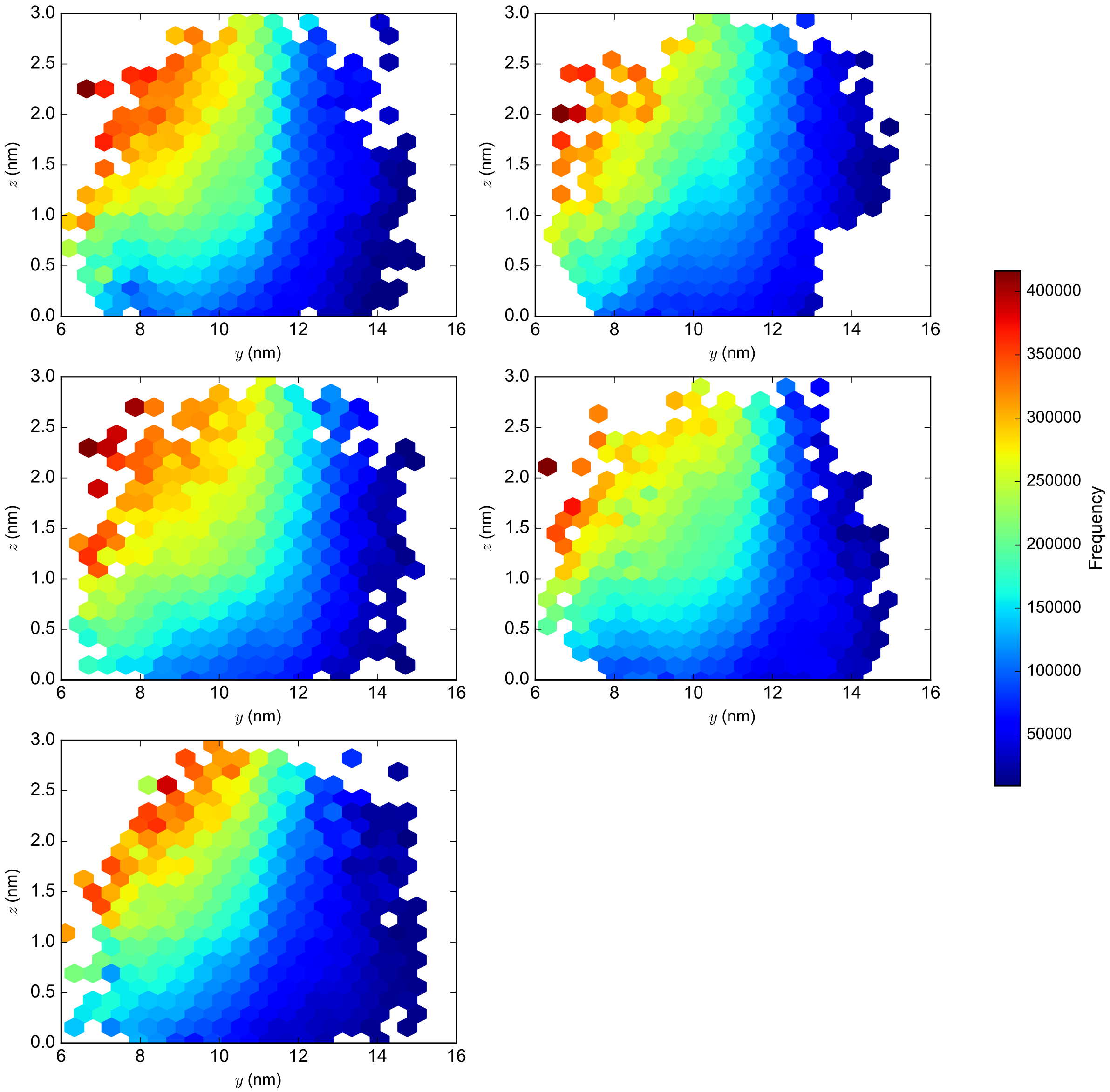

Figure S8: Spatial distribution of calculated local concentrations for five different datasets sampled using the UU model, each with a sample size of 10^4.

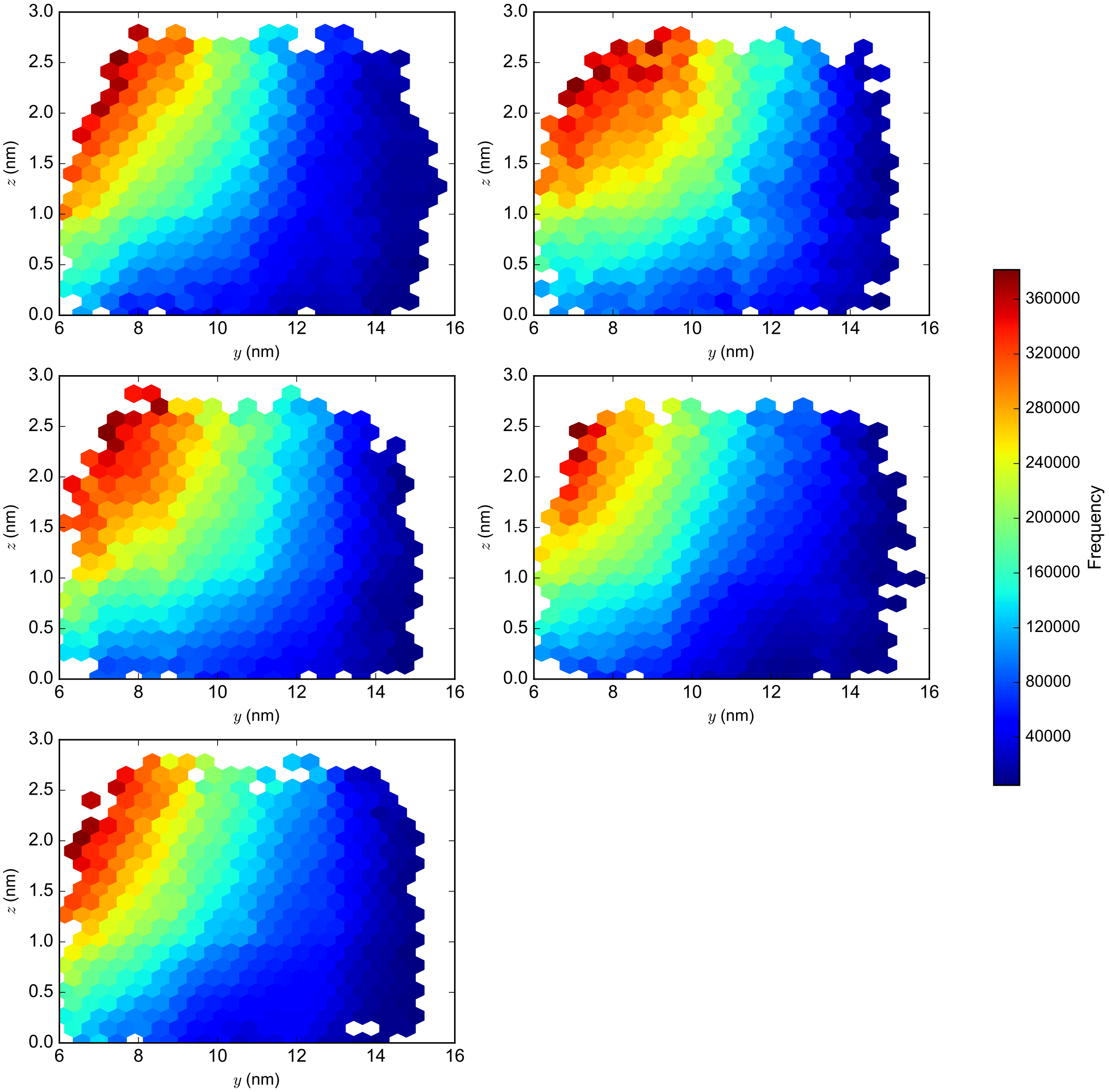

Figure S9: Spatial distribution of calculated local concentrations for five different datasets sampled using the WU model, each with a sample size of 10^4.

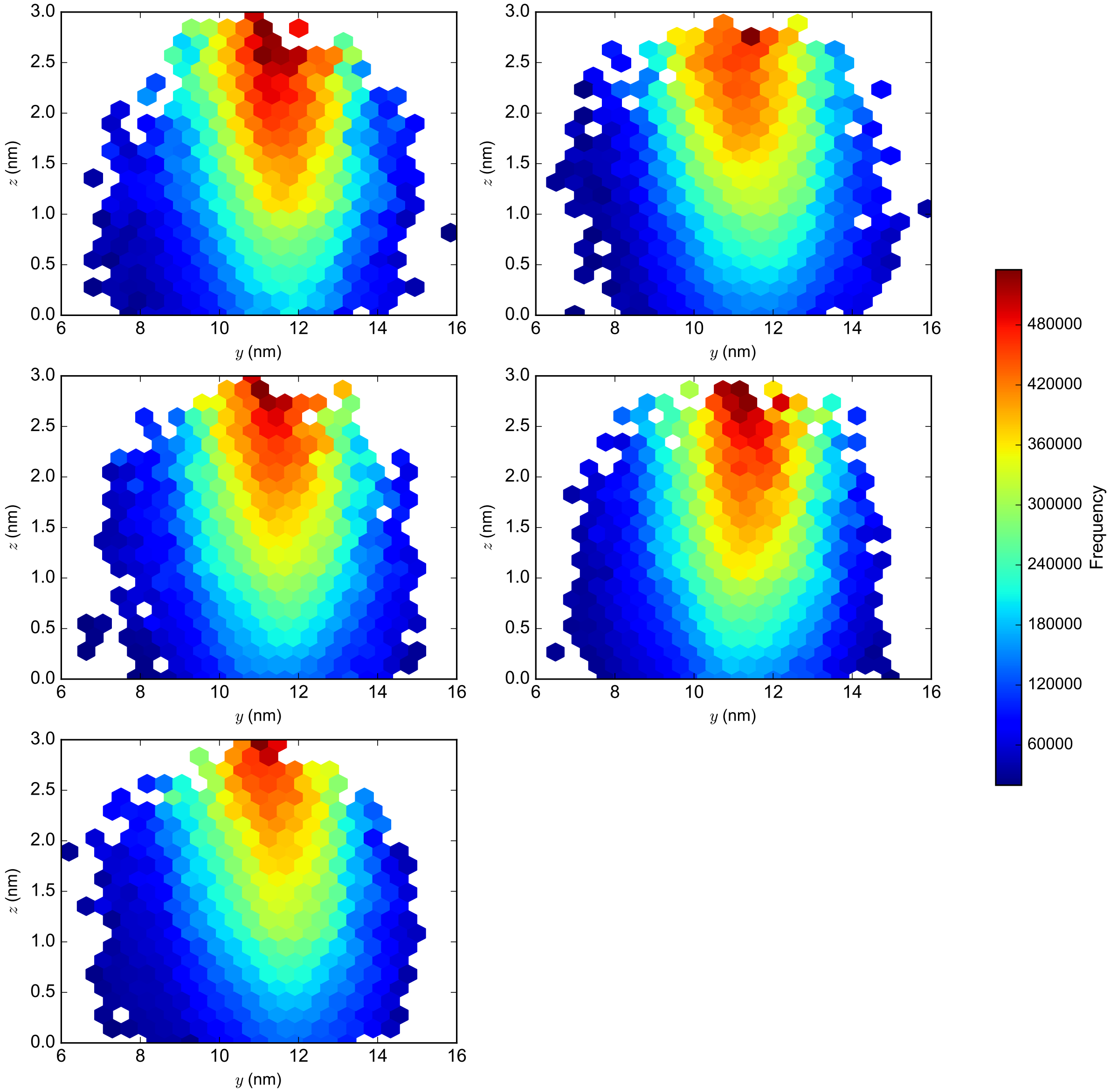

Figure S10: Spatial distribution of calculated local concentrations for five different datasets sampled using the UN model, each with a sample size of 10^4.

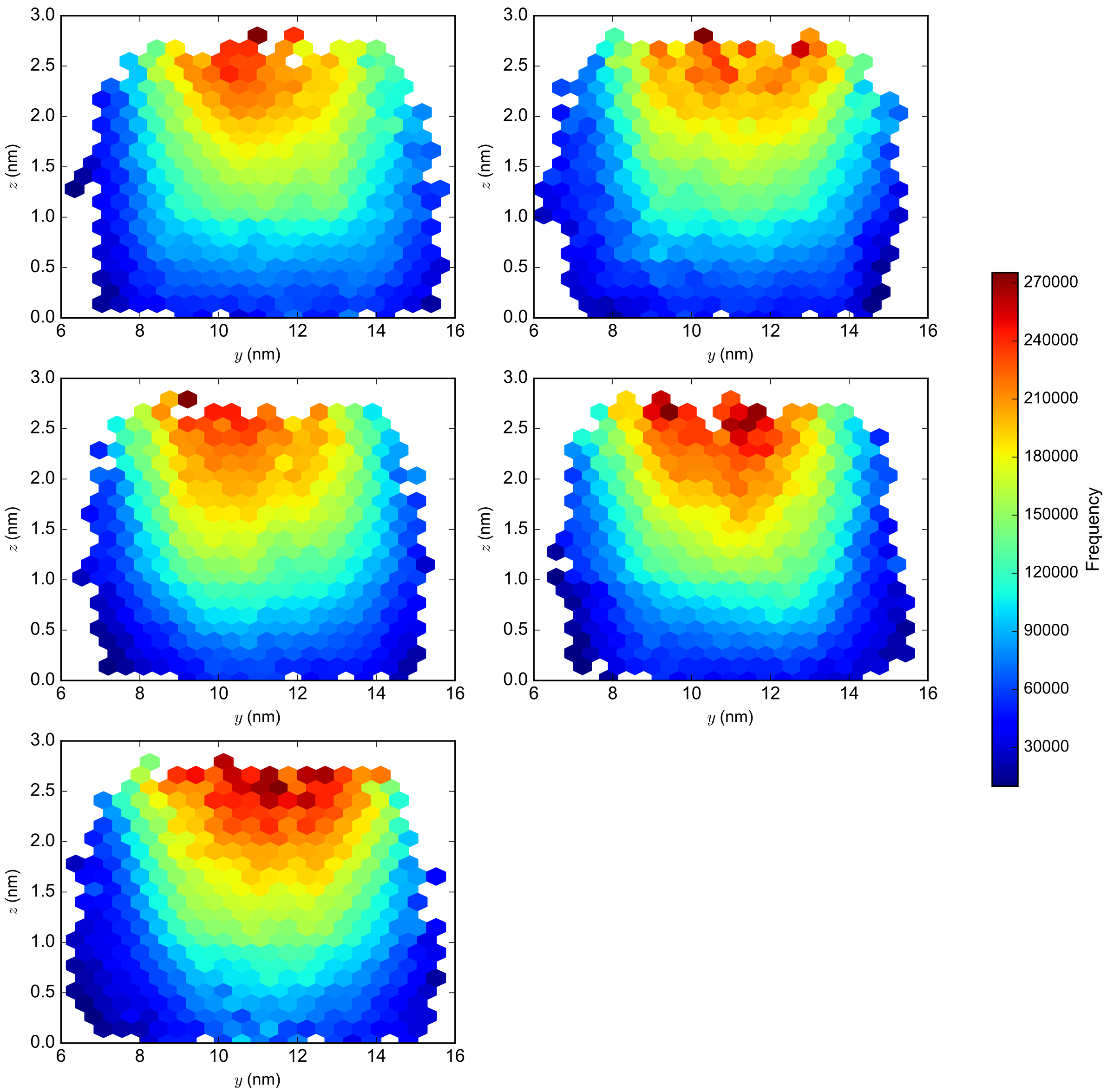

Figure S11: Spatial distribution of calculated local concentrations for five different datasets sampled using the WN model, each with a sample size of 10^4.

S4 Local concentration distribution for all models with 10^5 samples

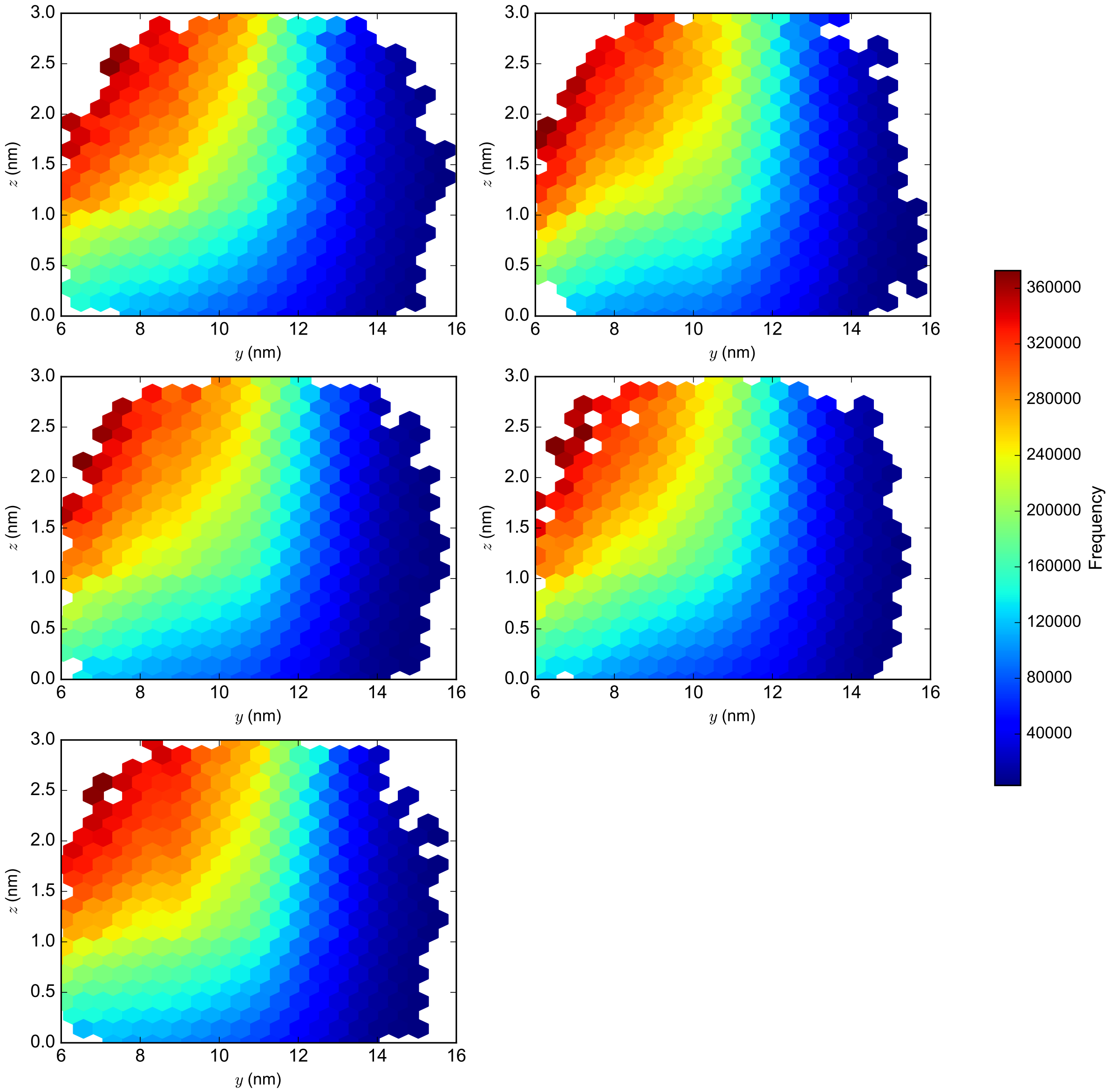

Figure S12: Spatial distribution of calculated local concentrations for five different datasets sampled using the UU model, each with a sample size of 10^5.

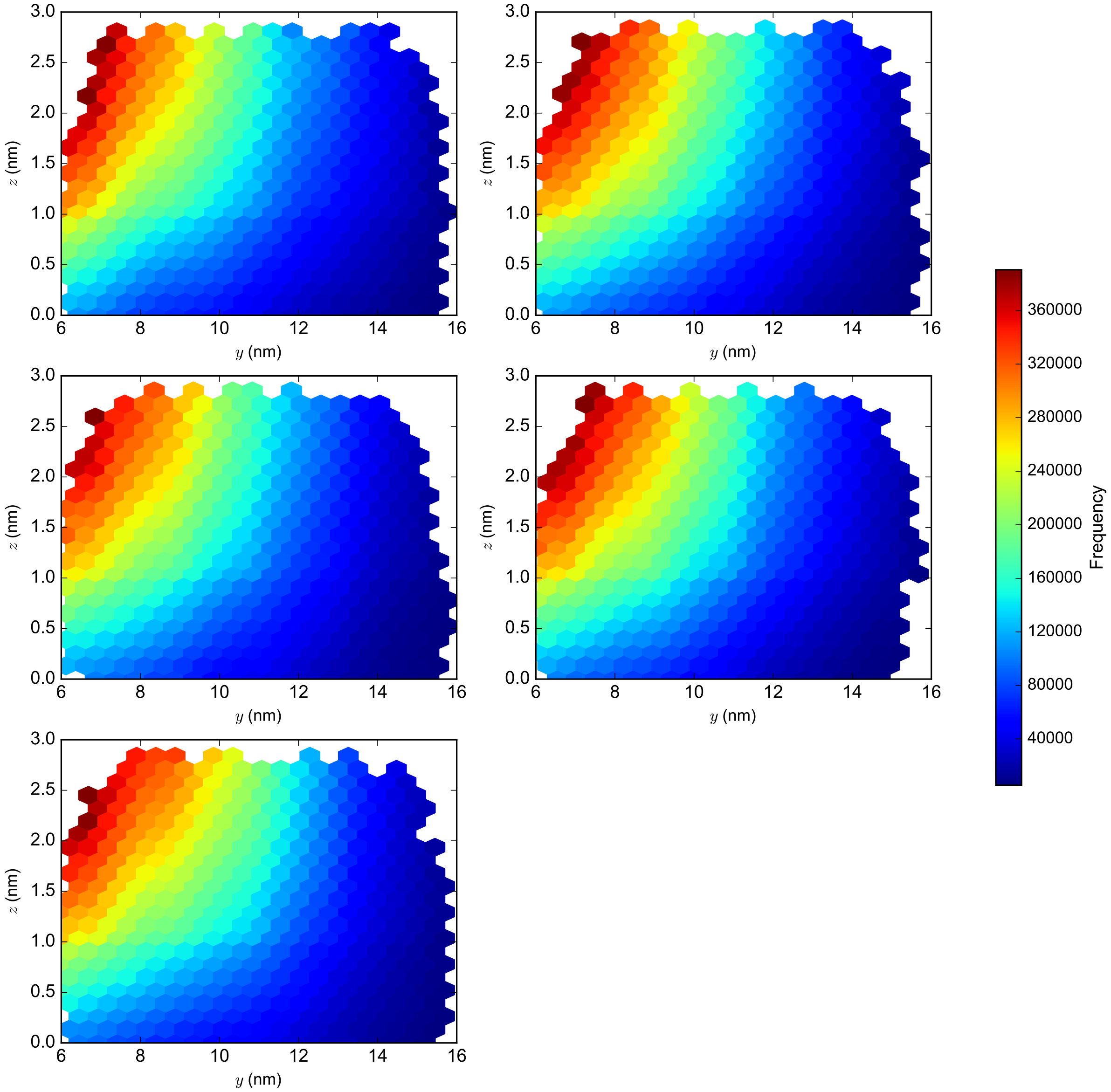

Figure S13: Spatial distribution of calculated local concentrations for five different datasets sampled using the WU model, each with a sample size of 10^5.

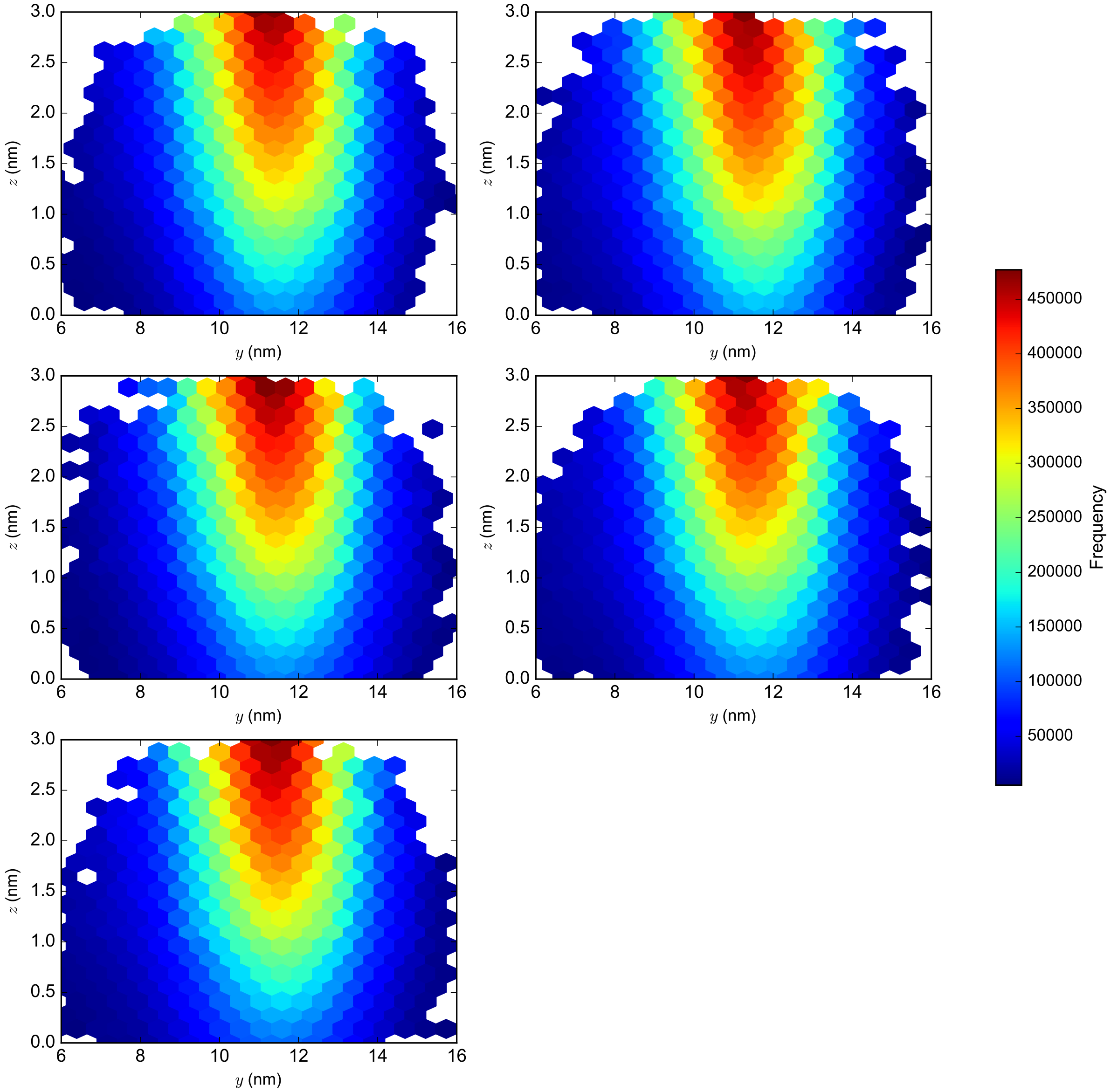

Figure S14: Spatial distribution of calculated local concentrations for five different datasets sampled using the UN model, each with a sample size of 10^5.

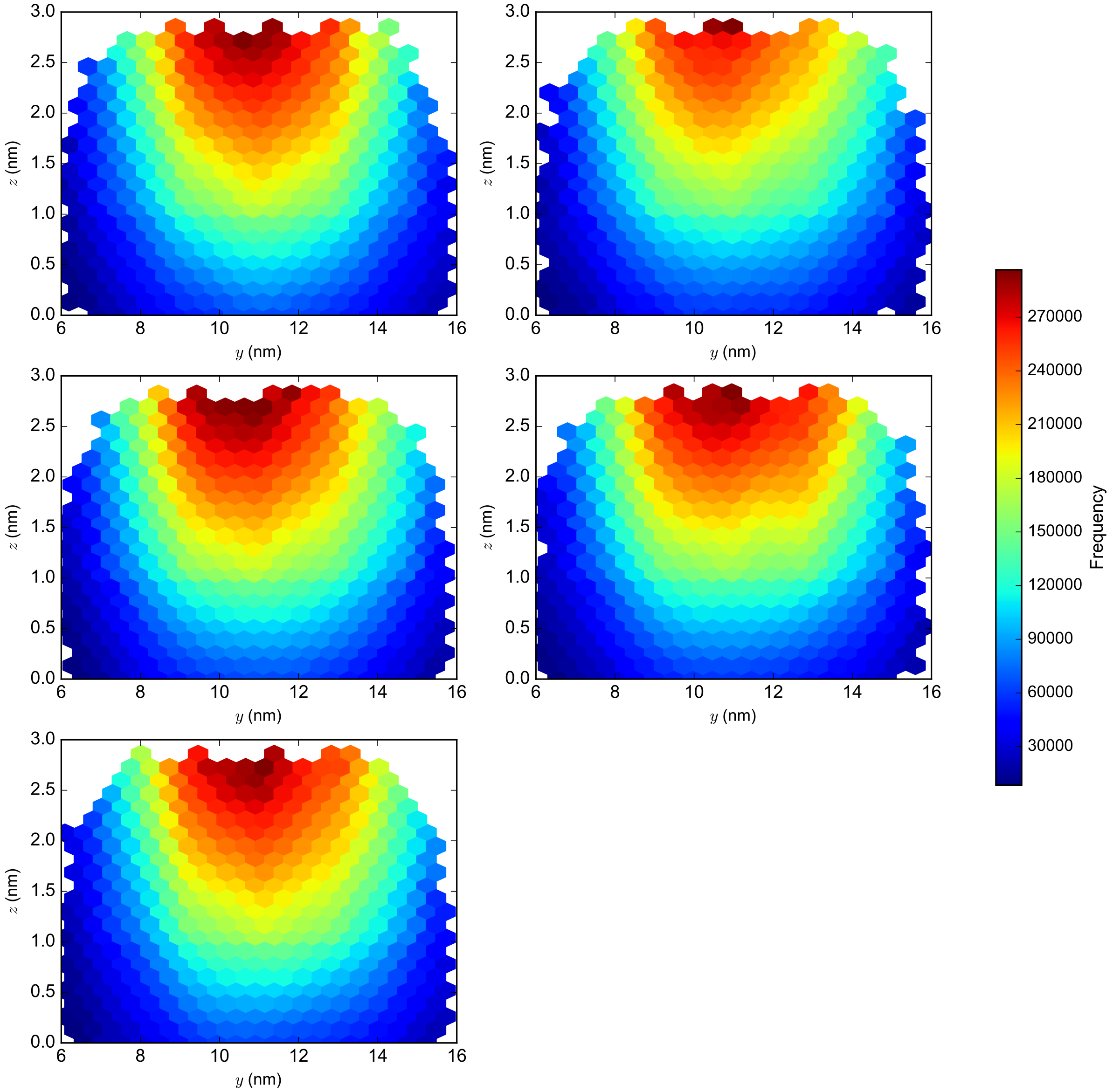

Figure S15: Spatial distribution of calculated local concentrations for five different datasets sampled using the WN model, each with a sample size of 10^5.

S5 Local concentration distribution for all models with 10^6 samples

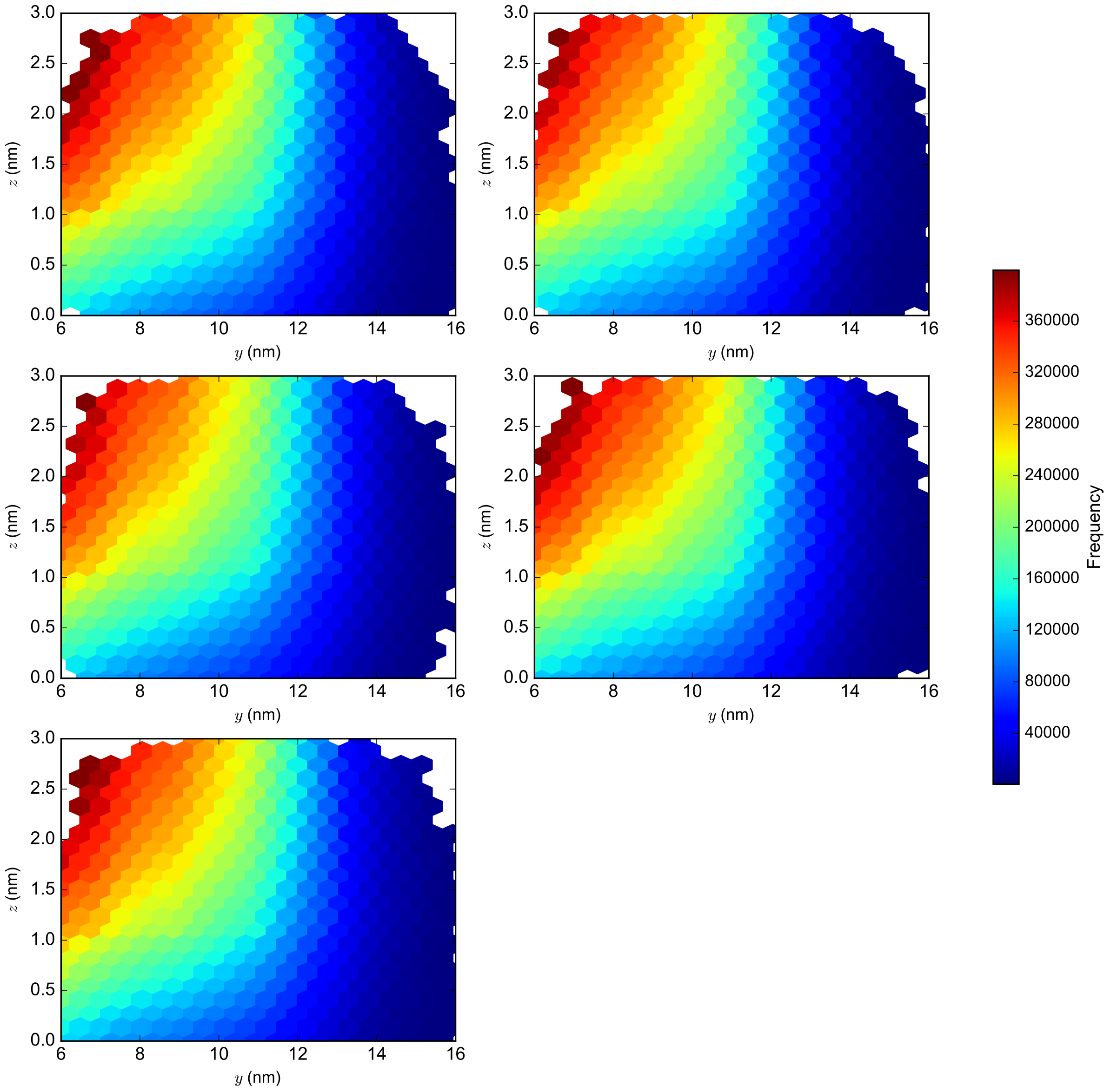

Figure S16: Spatial distribution of calculated local concentrations for five different datasets sampled using the UU model, each with a sample size of 10^6.

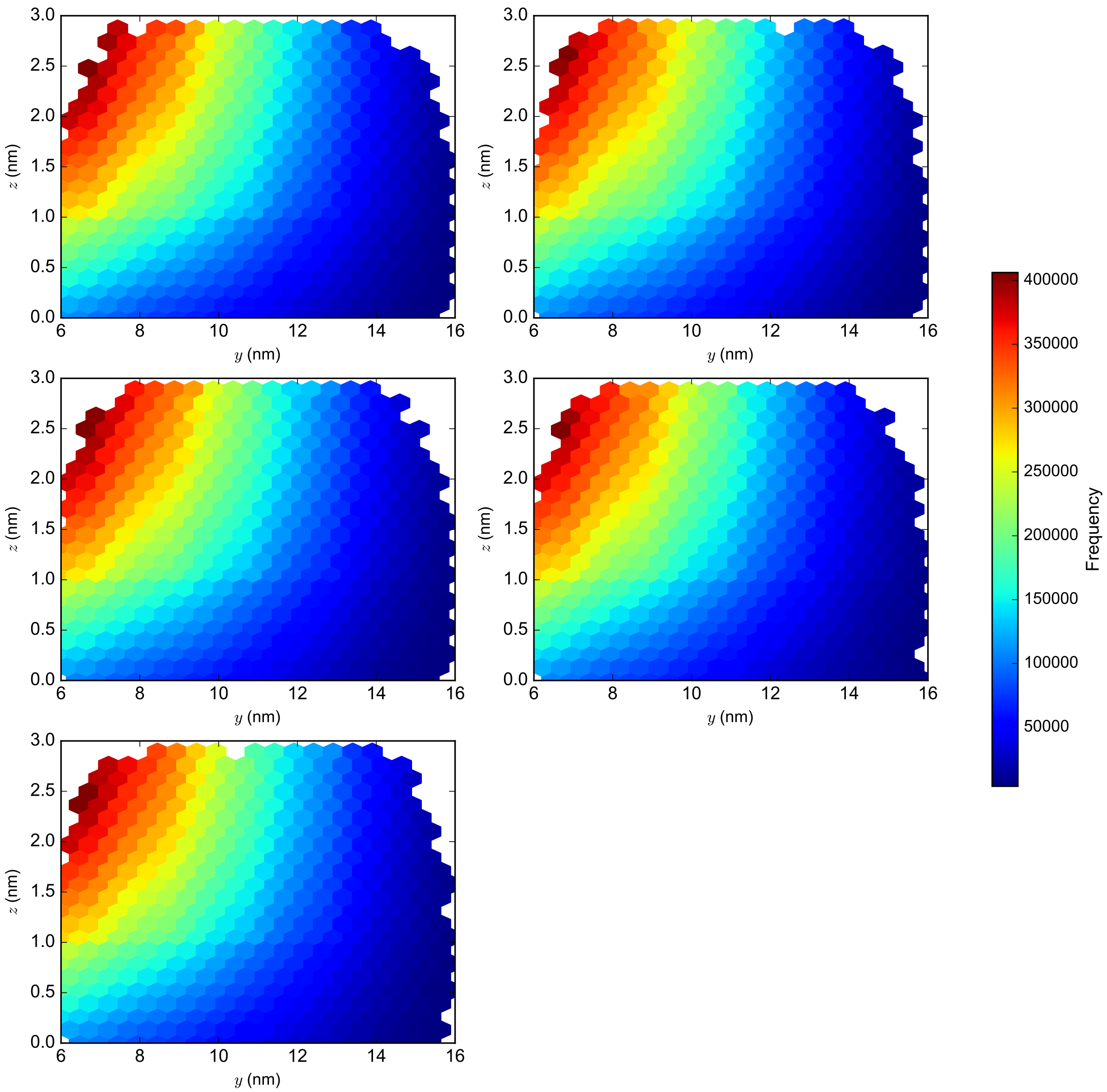

Figure S17: Spatial distribution of calculated local concentrations for five different datasets sampled using the WU model, each with a sample size of 10^6.

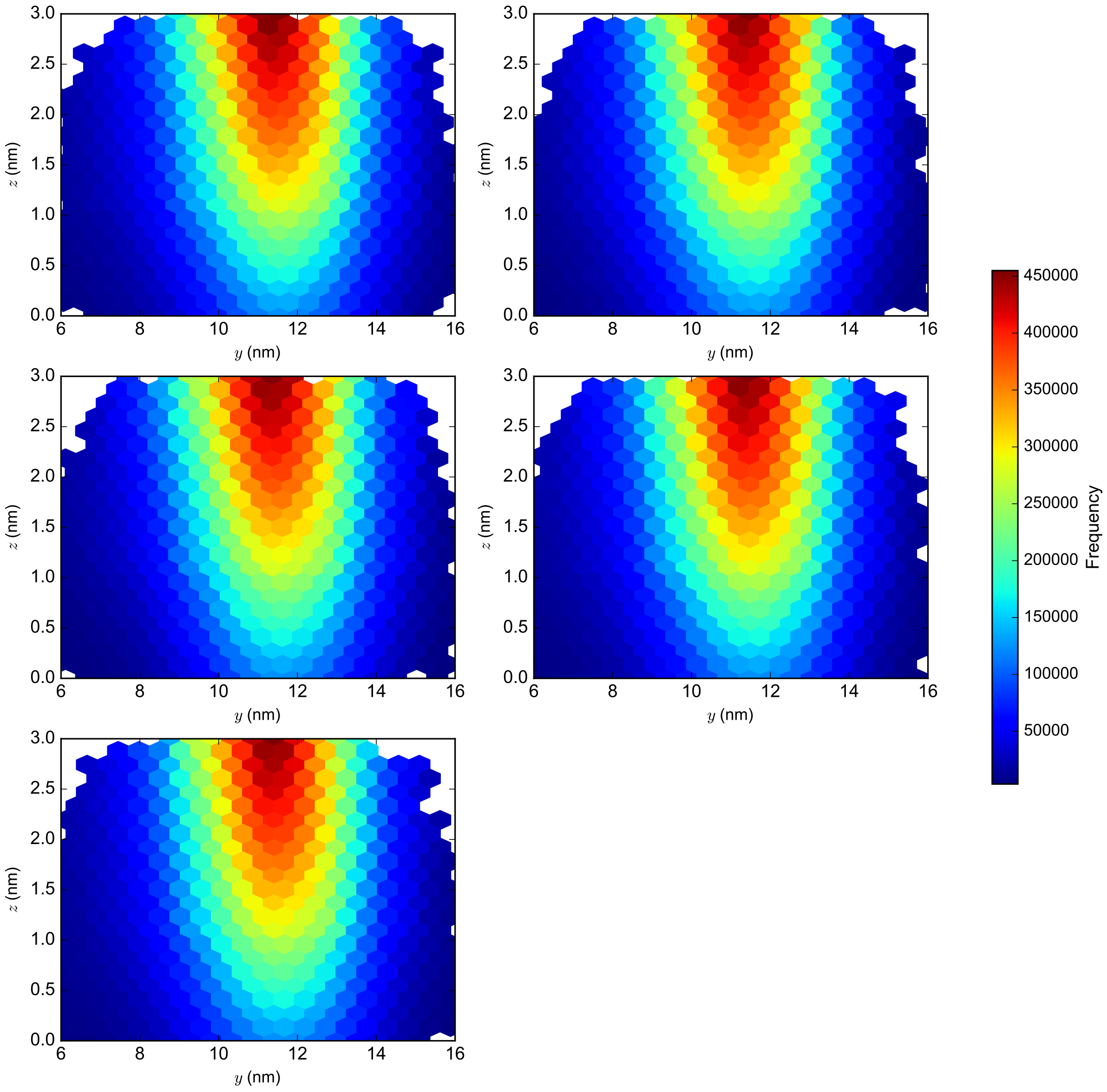

Figure S18: Spatial distribution of calculated local concentrations for five different datasets sampled using the UN model, each with a sample size of 10^6.

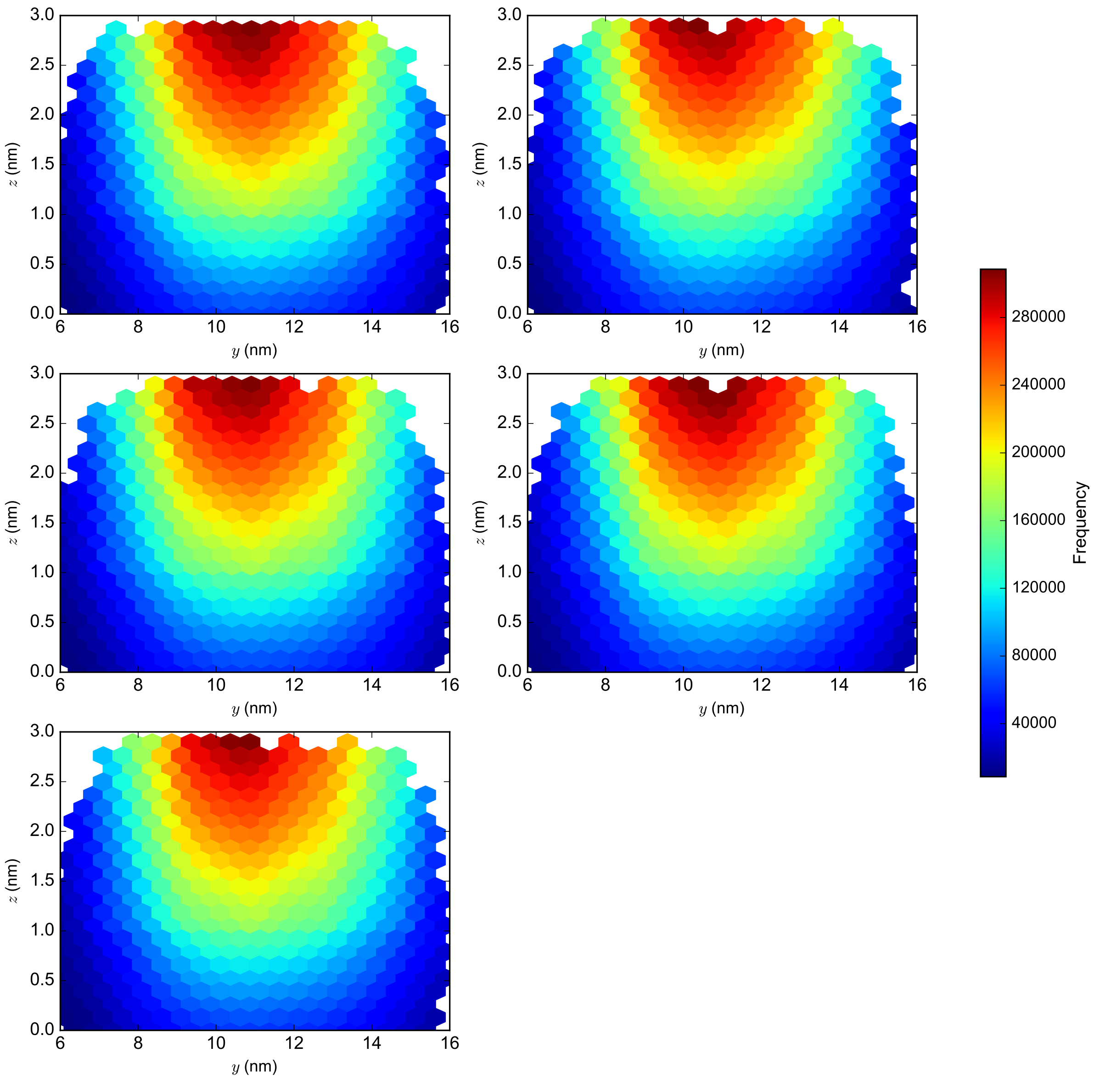

Figure S19: Spatial distribution of calculated local concentrations for five different datasets sampled using the WN model, each with a sample size of 10^6.

S6 Plots of overall local concentration against sample size

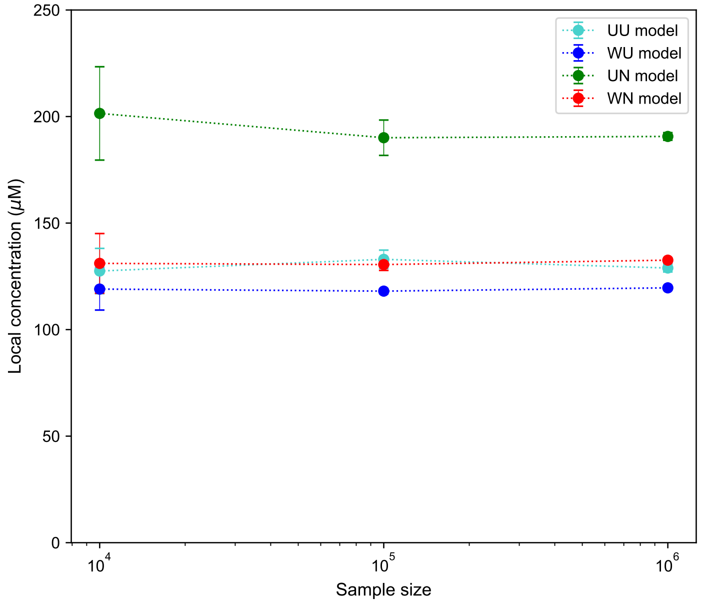

Figure S20: Inferred overall local concentrations for different sample sizes, for all four models (UU, WU, UN, WN) and with 10.88 nm distance between hairpins H_0 and H_1 and using a threshold distance of 2 nm between the reactive points to determine whether two structures may interact. Points and errorbars show mean and standard deviation of of five different datasets in each case.

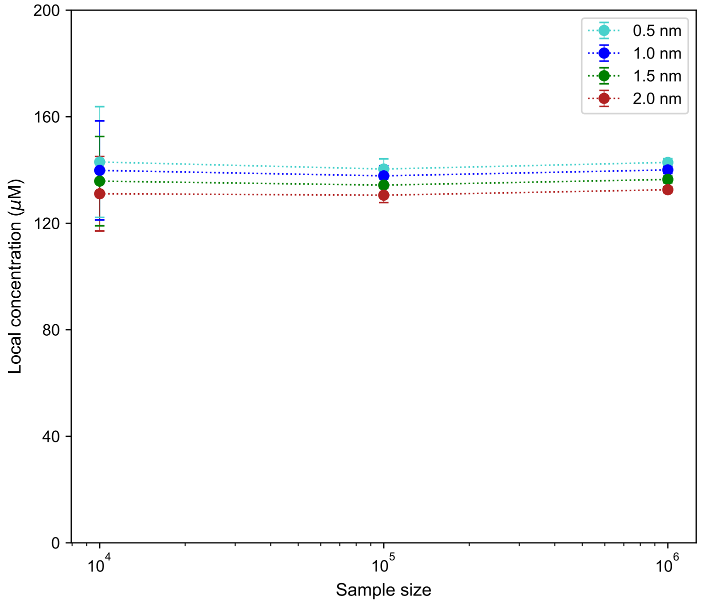

Figure S21: Inferred overall local concentrations for different sample sizes and different threshold lengths, for WN model and with 10.88 nm distance between hairpins H_0 and H_1. Points and errorbars show mean and standard deviation of of five different datasets in each case.

S7 Plot of overall local concentration against sample size for control variant with double-spaced hairpins

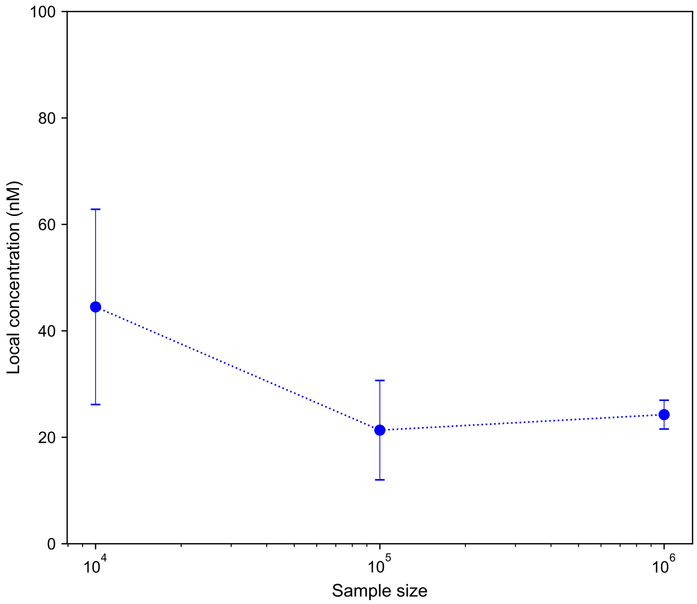

Figure S22: Inferred overall local concentrations for different sample sizes, for WN model and with double-spaced 21.76 nm distance between hairpins H_0 and H_1 and using a threshold distance of 2 nm between the reactive points to determine whether two structures may interact. **Note that the y-axis on this plot is in** nM **and not** μM **like the other plots of local concentration: in the double-spaced case, the local concentration is orders of magnitude smaller.** Points and errorbars show mean and standard deviation of of five different datasets in each case.

S8 Illustrative plots of non-uniform distributions used to parameterize the biophysical models

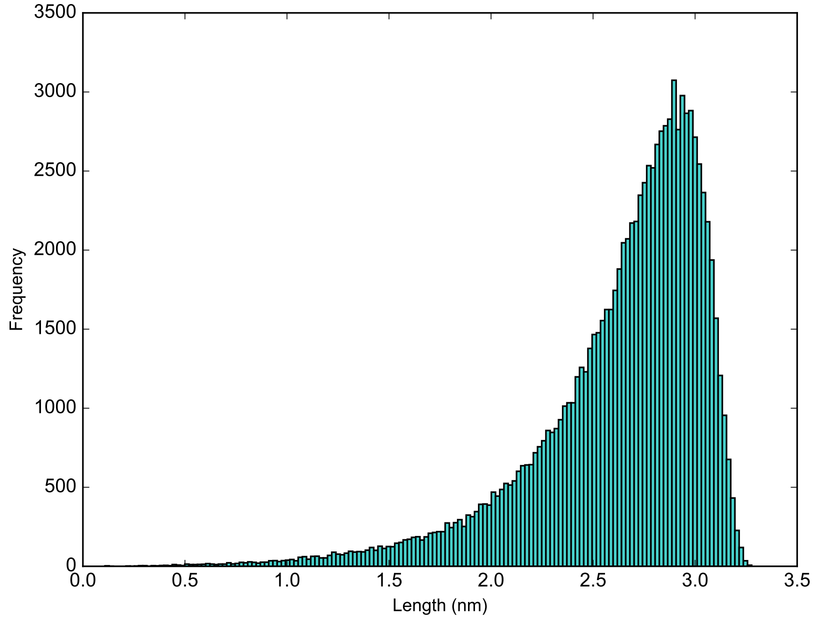

Figure S23: Example of a worm-like chain (WLC) distribution used for sampling lengths of single-stranded domains in our WU and WN models. This particular histogram shows the distribution of extended lengths for a 5-nucleotide single-stranded domain. We assume a length per nucleotide of 0.68 nm, giving an overall maximum extension $L = 3.4$ nm. We use $s = 2$ nm for the persistence length of single-stranded DNA.

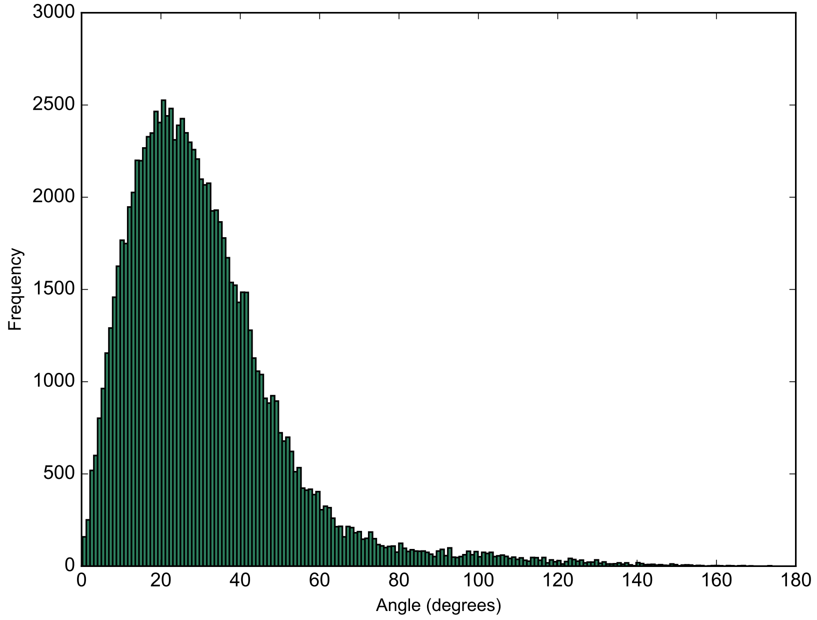

Figure S24: Nicked angle distribution computed by Chatterjee et al. [1], used to sample angles between double-stranded domains separated by a nick in our UN and WN models.

S9 Rejection sampling information

Table S1: Average of Rejection of the sampling data

	No. of samples desired	No. of samples discarded
UU model	1,000,000	4,375,891
WU model	1,000,000	6,538,448
UN model	1,000,000	3,095,698
WN model	1,000,000	5,374,627

S10 Modeling repulsive behaviour between DNA hairpins and DNA origami

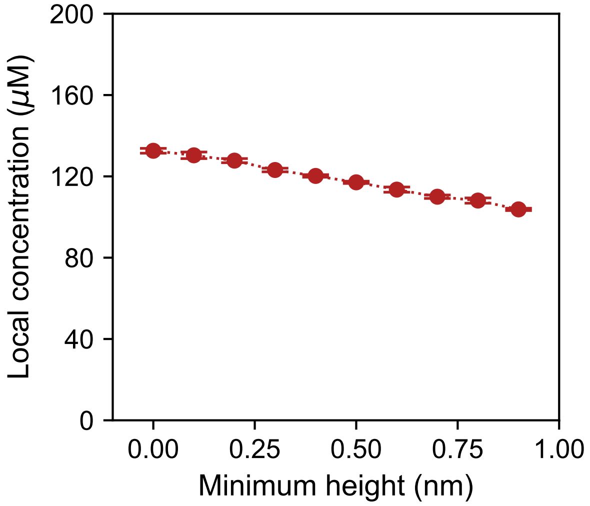

Figure S25: Inferred overall local concentrations for different minimum heights ϵ from the DNA surface (i.e. rejecting samples containing sampled coordinates where $z < \epsilon$). Simulations were carried out using the WN model with three different datasets having sample size 10^6, inter hairpin distance of 10.88 nm and using a threshold distance of 2 nm between the reactive points to determine whether two particular structures may interact. The local concentration value decreases linearly as the minimum height, i.e. ϵ value, increases. Points and errorbars show mean and standard deviation of of three different datasets in each case.

References

[1] Chatterjee G, Dalchau N, Muscat RA, Phillips A, Seelig G. A spatially localized architecture for fast and modular DNA computing. Nature Nanotechnology. 2017;12:920–927.
